# Assessing the decoupling of economic growth from environmental impacts in the European Union: A consumption-based approach

**DOI:** 10.1016/j.jclepro.2019.07.010

**Published:** 2019-11-01

**Authors:** E. Sanyé-Mengual, M. Secchi, S. Corrado, A. Beylot, S. Sala

**Affiliations:** European Commission, Joint Research Centre, Via Enrico Fermi 2749, I-21027, Ispra, Italy

**Keywords:** Environmental impact assessment, Life cycle assessment, Environmentally-extended input-output, Sustainable development goal 12, Decoupling, Sustainable development target 8.4

## Abstract

Pursuing a responsible and sustainable development, the United Nations urged to decouple economic growth from environmental impacts. Several European Union (EU) policies have been implemented towards such goal. Although multiple authors have evaluated the decoupling of the economic growth from the resource use or environmental concerns, the environmental assessment mostly focused on pressures rather than impacts, and used single indicators assumed to be a proxy of the overall effects on the environment. Furthermore, no studies were found using a process-based life cycle approach to quantify the environmental impacts of consumption. To solve such research gap, this paper assesses the decoupling in the EU focusing on potential environmental impacts, complementing a production-based approach with two options for accounting for the impacts of consumption. The aim of this paper is to evaluate the decoupling of the economic growth (in terms of Gross Domestic Product) from the environmental impacts due to EU-28 consumption, assessed by means of life cycle assessment (LCA). The decoupling is then assessed in impact terms rather than limited to pressures by using the Environmental Footprint (EF2017) indicators, which allows assessing 16 different impacts. The Consumption Footprint indicator quantified the environmental impacts of EU apparent consumption, including the territorial impacts (Domestic Footprint) and the embodied impacts in both imports and exports (Trade Footprint). The inventory of pressures for the trade component is compiled either with a bottom-up approach (process-based LCA of representative traded goods) or a top-down approach (input-output-based LCA). Methodological aspects influencing the decoupling assessment and the resulting outputs are presented and discussed. According to the results, the environmental impacts of EU-28 consumption showed decoupling during the last decades (2005–2014), between relative to absolute decoupling depending on the inventory modeling approach taken. Some countries showed higher decoupling levels than others displaying a heterogeneous map of EU-28 decoupling, which was led by acidification, particulate matter, land use and eutrophication impacts. Notwithstanding current limitations, the assessment of decoupling using consumption-based environmental indicators is very promising for supporting policy-making towards addressing the actual impacts driven by the EU production and consumption system.

## Highlights

• Comprehensive assessment of the decoupling of environmental impacts of EU-28 consumption from GDP.
• A life cycle perspective is taken for the environmental impact modeling.
• Decoupling assessment need further refinements to be used for policy support.
• Top-down and bottom-up approaches indicated from relative to absolute decoupling.
• Decoupling of environmental impacts from human development index is also evaluated.

## 1 Introduction

Since the “Our Common Future” report was published in the late 80s (Brundtland, 1987), the close and inverse relation between the progress of humankind and the global environmental quality has been placed on the spotlight. The inefficient economic growth of the last century increased the anthropogenic pressures to the environment, negatively affecting the global climate (IPCC, 2018) as well as the provision of ecosystem services (TEEB, 2011) leading humankind to transgress several “planetary boundaries” (Rockström et al., 2009; Steffen et al., 2015). Indeed, human development has brought several irreversible changes to the Earth system, determining that current geological age is called Anthropocene (Crutzen, 2002; Steffen et al., 2007). To tackle such unsustainable track, the United Nations (UN) developed as blueprint the Millennium Development Goals (UNDP, 2006) and, later, the Sustainable Development Goals (SDGs) (UN, 2015). The 17 SDGs address global challenges from food insecurity and poverty to innovation and climate action, with interconnected objectives and targets. Among them, achieving a sustainable production and consumption (SDG12) is one of the main goals towards facing environmental degradation and improving resource efficiency (UN, 2015). Moreover, target 8.4 of the SDGs seeks to “improve progressively, through 2030, global resource efficiency in consumption and production and endeavour to decouple economic growth from environmental degradation.”

In this context, the European Union (EU) adopted the 2030 UN Agenda for Sustainable Development and the SDGs to set the pathway for transforming EU production and consumption in “a low-carbon, climate resilient, resource efficient and circular economy” (EC, 2016a). Besides, EU policies such as the 7^th^ European Environment Action Program (7^th^ EAP) (EU, 2013) and the Roadmap to a Resource Efficient Europe (EC, 2011) are driving the achievement of environmental targets. More recently, the EU Circular Economy Action Plan (EC, 2015) and the Bio-Economy Strategy (EC, 2018) have represented a shift of policy-making towards acknowledging more and more the need of an integrated approach of production and consumption when addressing environmental impacts. A responsible and sustainable production and consumption (SCP) (SDG12) would contribute to a more resource-efficient Europe, as proposed in the Roadmap, with an improved resource productivity that decouples the economic growth from resource use and associated environmental impacts (Sala et al., 2016).

### 1.1 The decoupling of economic growth from resource depletion and environmental impacts as a sustainable pathway

**Fig. 1:**
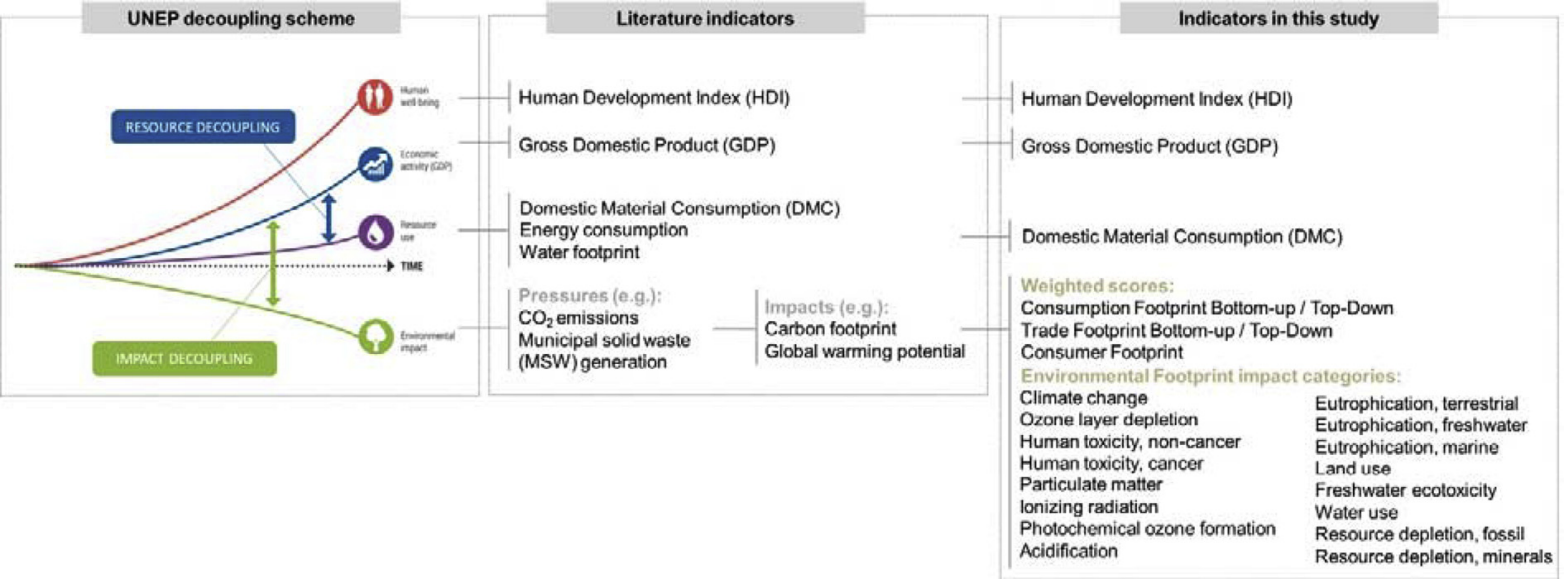
Decoupling scheme from IRP (2017), quantitative indicators employed in the literature and indicators proposed in this study.

Pursuing a more efficient development, the United Nations Environmental Program (UNEP) urged to decouple economic growth from environmental impacts (UNEP, 2011). Decoupling takes place “*when resource use or some environmental pressure either grows at a slower rate than the economic activity that is causing it (relative decoupling) or declines while the economic activity continues to grow (absolute decoupling)*” (IRP, 2017, p.23). Indeed, decoupling can take place at different levels: resource decoupling, environmental pressure decoupling or environmental impact decoupling (Fig. 1). Based on the specificity of the underpinning environmental processes, the environmental pressures (e.g. emission or resource use) may exert different impacts (e.g. the emission of two toxic substances may exert different ecotoxicity impacts). Hence, quantitative measures of decoupling may be performed by comparing the economic output (e.g. Gross Domestic Product, GDP) with indicators of resource use (e.g. Domestic Material Consumption, DMC), environmental pressures (e.g., CO_2_ emissions) and environmental impacts (e.g., global warming potential). Furthermore, UNEP indicated that human well-being progress should also decouple from the environmental impacts, in line with some authors that proposed using the Human Development Index (HDI) for assessing the progress of environmental policy (van den Bergh and Botzen, 2018) or evaluated human well-being indicators against planetary boundaries to contrast human basic needs and global resource use (O'Neill et al., 2018).

Both resource and environmental decoupling have been largely evaluated in the literature, as detailed in the collection of studies in Table 1. While some authors kept a production approach where domestic and territorial flows are considered, a consumption-based approach is key for integrating the trade flows into the decoupling assessment, as embodied impacts due to imports usually displaced to developing countries can greatly contribute to the overall environmental impacts of consumption (Hamilton et al., 2018; Wiedmann and Lenzen, 2018; Wood et al., 2018). The geographical scope of the studies in literature ranged from global (Bithas and Kalimeris, 2013; Schandl et al., 2016) to specific economic sectors (Li et al., 2017). Decoupling assessment was also employed to determine the efficacy of policy implementation, e.g. the Kyoto Protocol in Europe (Diakoulaki and Mandaraka, 2007) and in China (Wang and Yang, 2015).

**Table 1:** Details of collected decoupling studies by decoupling type (resource – R, environmental – E) and approach (production – P, consumption – C), geographical area, timeframe, resources/environment (R/E) accounting and indicator, and economic indicator.

Decoupling		Geography	Timeframe	R/E accounting	R/E indicator	Economic indicator	Study
Type	Approach						
R	P,C	United States	1870–2005	Economy-wide MFA	Domestic Material Consumption (DMC) Total Primary Energy Supply (TPES)	GDP Income (GDP per capita)	[1]
R	C	Worldwide	1980–2009	Economy-wide MFA	DMC Material productivity	GDP	[2]
R	C	Worldwide World regions Countries	1900–2009	Economy-wide MFA	DMC	GDP Income (GDP per capita)	[3]
R	C	Worldwide	1900–2009	Economy-wide MFA	Energy use	GDP Income (GDP per capita)	[4]
E	C	Beijing – Tianjin – Hebei, China	1996–2010	National accounting	Energy consumption CO_2_ emissions (IPCC)	GDP	[5]
E	C	Jiangsu, China	1995–2009	Energy accounting (EFA)	Energy-related CO_2_ emissions (IPCC)	GDP	[6]
E	C	Brazil	2004–2009	National energy balance (EFA)	Energy-related CO_2_ emissions (IPCC)	GDP	[7]
E	C	European Union (EU25)	1995–2005	National accounting	Municipal Solid waste (MSW)	Final consumption expenditure	[8]
E	C	Taiwan; Japan; Germany; South Korea	1990–2002	National accounting (OECD)	Highway transport-related CO_2_ emissions (IPCC)	GDP	[9]
E	C	Gulf countries	1980–2010	National accounting	Energy consumption Energy-related CO_2_ emissions (IPCC)	Income (GDP per capita)	[10]
E	C	China (textile industry)	2001–2014	National statistics	Water footprint	Gross annual industrial output	[11]
E	P,C	EU countries	1993–2010	National accounting	Carbon footprint	GDP	[12]
E	C	EU manufacturing sector	1990–2003	Energy accounting	Energy-related CO_2_ emissions (IPCC)	Total added value	[13]
E	C	Italy (energy sector)	1998–2006	National accounting (MFA)	Energy consumption CO_2_ emissions (IPCC)	GDP	[14]
R, E	P, C	World	2010–2050	Economy-wide MFA	Material intensity Energy intensity Carbon emissions	Income (GDP per capita)	[15]
R,E	C	China	1978–2010	Economy-wide MFA	Domestic extraction used (DEU) Total energy consumption CO_2_ emissions SO_2_ emissions Soot emissions Wastewater emissions COD emissions NH_3_ emissions	GDP	[16]
R, E	C	Macao, China	2000–2013	Greenhouse gas protocol	Total energy consumption (TEC) Total embodied GHG emissions (TEGE)	GDP	[17]
R, E	C	World 1995–2011	1995–2011	Multi-regional input-output	GHG emissions Energy use Material use Blue water consumption Land use	GDP	[18]
R,E	C	Scandinavia (industry)	1993–2001	Energy accounting	Energy consumption Energy-related CO_2_ emissions	Gross added value	[19]

^[1]^(Gierlinger and Krausmann, 2012); ^[2]^(Giljum et al., 2014); ^[3]^(Bithas and Kalimeris, 2018); ^[4]^(Bithas and Kalimeris, 2018); ^[5]^(Wang and Yang, 2015); ^[6]^(Zhang and Wang, 2013); ^[7]^(de Freitas and Kaneko, 2011); ^[8]^(Mazzanti and Zoboli, 2008); ^[9]^(Lu et al., 2007); ^[10]^(Salahuddin and Gow, 2014); ^[11]^(Li et al., 2017); ^[12]^(Liobikiene and Dagiliute, 2016)^[13]^(Diakoulaki and Mandaraka, 2007); ^[14]^(Andreoni and Galmarini, 2012); ^[15]^(Schandl et al., 2016); ^[16]^(Yu et al., 2013); ^[17]^(Chen et al., 2017); ^[18]^(Wood et al., 2018); ^[19]^(Enevoldsen et al., 2007).

At the global level, Giljum et al. (2014) stressed the need to focus on guaranteeing an absolute decoupling (i.e., decreasing trend of environmental pressures or impacts) that approaches dematerialization and green economy since relative decoupling (i.e., increasing trend of environmental pressures or impacts even if at lower pace compared to GDP) is linked to an increased resource consumption, even though the achieved higher material productivity during the last decades. Furthermore, assessing environmental impact decoupling is essential to determine how environmental policy affects the environmental quality beyond resource use intensity. Although some studies evaluated environmental decoupling, the assessment was usually focusing on environmental pressures only, namely addressing specific emissions, e.g., CO_2_ emissions (Chen et al., 2017; Gierlinger and Krausmann, 2012), or specific economic sectors, e.g., energy (de Freitas and Kaneko, 2011; Wang and Yang, 2015; Zhang and Wang, 2013). Therefore, environmental decoupling was mainly studied for specific indicators and environmental pressures (i.e., energy consumption, CO_2_ emissions) assuming to be a proxy of the overall effects on the environment (e.g., acidification, eutrophication, toxicity). However, addressing environmental impacts is crucial to comprehensively assess environmental decoupling. In the reviewed studies, the economic output was the driving force originating the environmental pressure: GDP (Chen et al., 2017; Gierlinger and Krausmann, 2012), income (GDP per capita) (Bithas and Kalimeris, 2018; Salahuddin and Gow, 2014; Schandl et al., 2016), or added value (when assessing specific sectors, e.g. industry) (Enevoldsen et al., 2007; Li et al., 2017). Finally, most of the studies quantified the environmental pressures through national statistics and Material Flow Analysis (MFA). Multi-regional input-output (MRIO) with environmental extensions (e.g., Exiobase) (Merciai and Schmidt, 2017; Stadler et al., 2018; Wood et al., 2015) as well as the Greenhouse gas protocol (WBCSD, 2004) were also employed in the literature. However, no studies were found using a process-based life cycle assessment (LCA)-based approach to quantify the environmental pressures or impacts in the decoupling assessment.

In this context, this paper contributes to the decoupling literature in a number ofaspects:

• The decoupling assessment is performed at both production (territorial/domestic) level and consumption level. The paper evaluates the decoupling of the environmental impacts of consumption in the European Union from GDP.
• The environmental assessment is performed in terms of environmental impacts rather than limited to pressures. Multiple impact indicators are used, adopting the Environmental Footprint impact categories and models (EC, 2013). Impact indicators are also normalized and weighted in a single score footprint.
• The decoupling assessment is complemented by the evaluation of the decoupling of the HDI from the environmental impacts (Fig. 1) to address aspects which are going beyond the mere economic growth assessed by GDP.
• The influence of methodological decisions is addressed to unveil the effect of inventory modeling and normalization.
• The consumption impacts are modelled, adding to the production impacts those of import and subtracting those of export. The trade footprint is calculated adopting two alternative approaches: a bottom-up approach (process-based LCA of representative traded goods) and a top-down approach (input-output-based LCA related to trade).

### 1.2 Goal and objectives

The goal of this paper is to evaluate the decoupling of the economic growth (in terms of GDP) from the environmental impacts of EU-28 consumption by employing LCA-based environmental indicators and exploring the methodological aspects that influence the decoupling assessment and resulting outputs. Specific objectives aim to address the following research questions:

- How has GDP decoupled from the environmental impacts of EU-28 consumption in the timeframe (2005–2014)? How is the environmental decoupling taking place in the different EU countries in the timeframe (2004–2011)?

- Which are the environmental impact categories driving the decoupling? How have the EU environmental policies influenced the decoupling from environmental impacts?
- To what extent does life cycle modeling and impact assessment affect the assessment of decoupling?
- Which are the environmental issues missing in the current assessment?
- How can decoupling be assessed beyond GDP?

## 2 Material and methods

The decoupling of GDP from environmental impacts due to EU-28 consumption was evaluated through the decoupling index (DI). The environmental impacts were quantified by adopting the LCA-based Consumption Footprint (CF) set of indicators (Sala et al., 2019a, Sala et al., 2019b). The CF adopts the impact categories recommended in the Environmental Footprint (EF) life cycle impact assessment (LCIA) method(EC, 2013). The following sections detail the calculation principles of the DI and the CF. Moreover, the resource decoupling has been as well performed to illustrate how this differs from the decoupling from environmental impacts. Finally, the sections presents the evaluation of decoupling beyond GDP by using the HDI indicator.

### 2.1 DDecoupling index

The decoupling can evaluate how resource use or environmental impact are linked to the economy by calculating a specific index, such as proposed by Tapio (2005) with the calculation of the elasticity between the changes along time of the two assessed parameters, e.g. transport volume and GDP. This ratio was later coined as Decoupling Index (DI) (UNEP, 2011), which has further been used in the literature (e.g., Wu et al., 2018). In this study, the DI is the ratio between the relative variation of environmental impacts (EI) and the relative variation of the economic output, in terms of GDP (Eurostat, 2018), for a defined timeframe.

DI=ΔEIΔGDP=(EIt+1−EIt)EIt(GDPt+1−GDPt)GDPt

The environmental decoupling was classified in:

- Absolute decoupling: DI < 0, ΔEI<0, ΔGDP>0. The environmental impact decreases while the economic output keeps growing.
- Relative decoupling: 0<DI<1, ΔEI>0, ΔGDP>0. The environmental impact increases although at a slower pace than the economic output.
- Non-decoupling: DI > 1, ΔEI > 0, ΔGDP>0. The environmental impact increases more than the economic growth.
- Stagnant: DI≠0, ΔEI<±0.1, ΔGDP≤0. The economic output remains stable or decreases while the environmental impact shows a small variation, suggesting that the decoupling is linked to the stagnation of the economy.

The classification has been adjusted from the one proposed by Wu et al. (2018) by including the category “stagnant”, which reflects e.g. the situation of some EU Member States with no economic growth or recession. The variation of environmental impacts to define a decoupling trend as “stagnant” (i.e., ΔEI<±0.1) was determined according to the observed trends in EU Member States with no economic growth or recession (i.e., Italy, Greece).

### 2.2 Consumption footprint: how the environmental impacts are assessed

The Consumption Footprint (CF) (Sala et al., 2019a) is an LCA-based set of indicators aiming at tracking the overall environmental impacts of consumption in the EU-28. LCA was chosen as the method for calculating the CF indicator due to its comprehensiveness, which allows not only assessing impacts along supply chains and diverse potential environmental impacts but also unveiling potential trade-offs (i.e. burden shifting from one environmental impact category to another or between different stages of the life cycle of products (Sala et al., 2016)).

The CF takes into account both the impacts associated with domestic activities (within the territorial boundaries of EU) (Domestic footprint) and the ones associated with trade (i.e. embedded impacts of imports and exports) (Import and Export footprints).

The CF is calculated as:

Consumptionfootprint=Importfootprint+Domesticfootprint−Exportfootprint

**Fig. 2:**
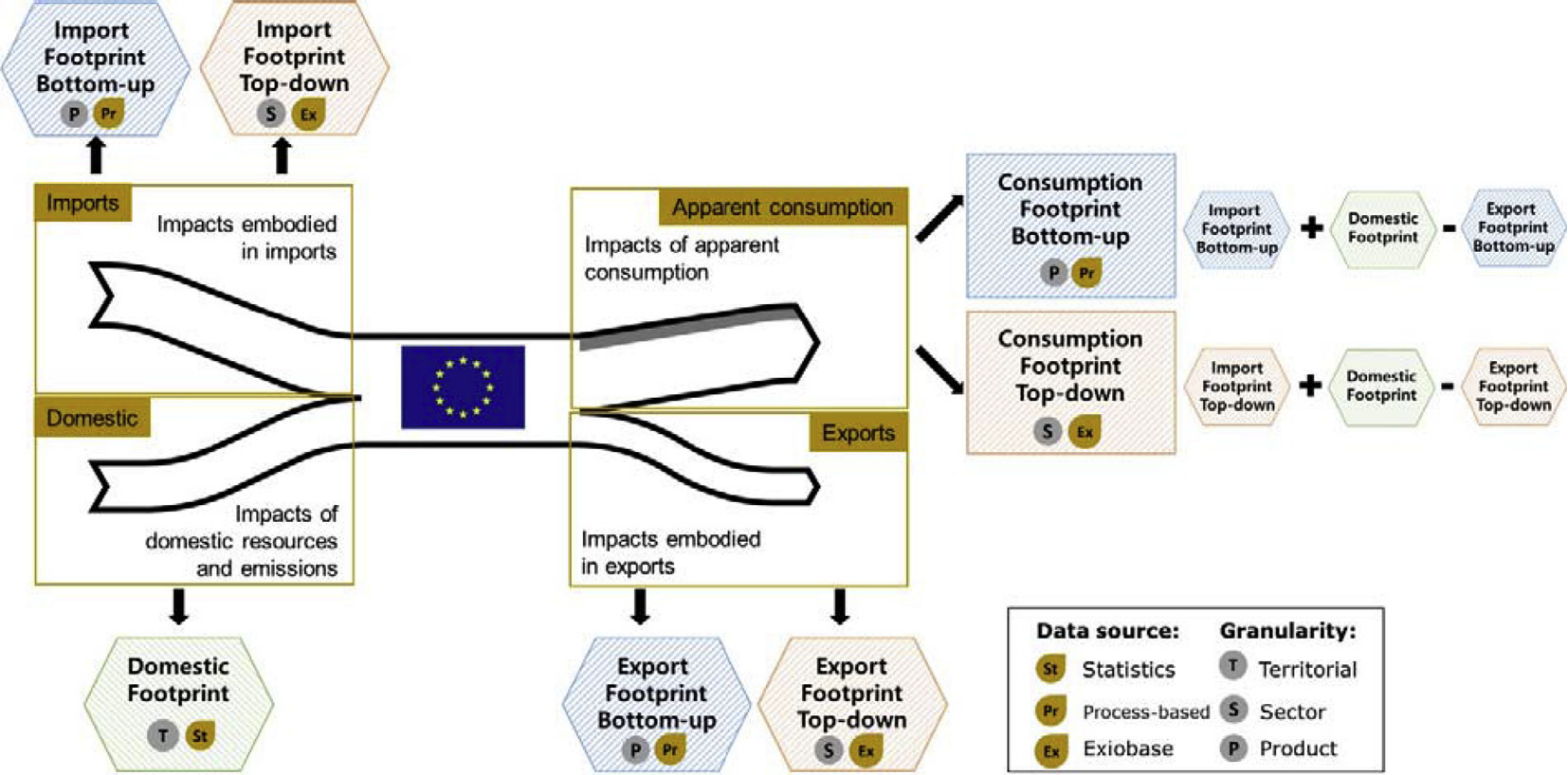
Scheme of the consumption elements, data sources and level of detail of the Consumption footprint bottom-up (CF-BU) and top-down (CF-TD), Domestic footprint (DF) and trade footprints. Apparent consumption differentiates household consumption (white) and the consumption from governments and non-profit institutions (grey).

The Domestic Footprint (DF) (Fig. 2) quantifies impacts stemming from both production and consumption activities taking place within the Member State's territory. This means that environmental impacts occurring in EU due to those activities comprised under economic sectors such as industry, agriculture, energy, mining and services were accounted for as ‘domestic’ component. Similarly, environmental impacts stemming from households and government's activities, such as food consumption or touristic services, were included as well in the ‘domestic’ component as they occur within the boundaries of a country.

Concerning the trade component of the CF, two approaches were followed to build the inventory of emissions and resources related to import and export (Fig. 2):

• Consumption Footprint Bottom-up (CF-BU): the DF was combined with trade impacts calculated based on the process-based LCA (ISO, 2006a, 2006b) of 40 selected representative products of the most imported products in and exported ones from EU-28 (Sala et al., 2019a).
• Consumption Footprint Top-down (CF-TD): the DF was combined with trade impacts calculated using sectorial-based Multi-Regional Input-Output Tables (MRIOTs), considering the Exiobase database version 3 (Beylot et al., 2019; Merciai and Schmidt, 2017; Stadler et al., 2018; Wood et al., 2015).

#### 2.2.1 Inventory of environmental pressures: resource use and emissions

This section details the compilation of the inventory data for the DF, CF-BU and CF-TD.

Domestic Footprint (DF). The DF relied on a vast inventory consisting of data on emissions and resource extraction and use in EU-28 territory, as detailed in Sala et al., 2015 and Sala et al. 2019b. The inventory is built gathering data from: i) official statistics at national, European or international level (e.g., United Nations Convention on Climate Change (UNCCC), Eurostat, the Food and Agricultural Organization (FAO) of the United Nations, the Organisation for Economic Co-operation and Development (OECD)); ii) emission models, which estimate emissions based on proxy indicators (Sala et al., 2019a). The final inventory is composed by: (i) raw data, as provided by third parties (e.g., greenhouse gas emissions in UNFCCC reports); (ii) estimated data from emission models (e.g., total nitrogen and phosphorus emissions to water from wastewater treatment plants); (iii) extrapolated data, following a gaps filling procedure to complement original data sources. The DF compiled statistical data for up to 1,957 environmental flows for the EU. At the MS level, the number of flows considered depended on the specific country.

Consumption Footprint Bottom-up (CF-BU) in which the Trade is based on Bottom-up Footprints. The CF-BU built upon the above-described DF, combined with import and export inventories based on process-based LCA of representative products of traded goods selected from Comext statistics (Eurostat, 2017). The up-scale procedure adopted to complete the final inventory was based on 40 products that represented the most imported and exported product groups (according to the two-digits Harmonised System nomenclature) in terms of mass and economic value in the respective year (Sala et al., 2019b). Around 1,500 environmental flows were individuated in the trade component of the CF-BU.

Consumption Footprint Top - Down(CF-TD) in which the Trade is based on Top-Down Footprints. The CF-TD built upon the above-described DF, combined with import and export inventories calculated using sectorial-based and environmentally-extended MRIOTs (Beylot et al., 2019). The hybrid version of Exiobase 3 was used to retrieve inventory data related to imports to and exports from EU-28 by differentiating the 28 EU Member States and 113 products and services. Additionally, all the results included investments (usually referred to as “capital goods” or “infrastructure” in LCA) attributed to import and export, since they were integrated within the MRIOTs. The environmental extensions distinguished 78 elementary flows: 36 mineral, metal and energy resources, 5 land occupation types, 3 water consumption types, and 29 substances emitted to air, 2 to water and 3 to soil.

#### 2.2.2 Impact assessment: characterization, normalization and weighting

The EF LCIA (EF2017) method (EC, 2017) was adopted for the impact assessment phase. The assumptions made to map the environmental data of statistical sources (DF) or the emissions and resources of the environmental extensions of Exiobase v3 (CF-TD) with the list of elementary flows of the LCIA method applied, i.e. EF2017, was a key aspect in the quantification of the environmental impacts. Beylot et al. (2019) details this mapping for the CF-TD. The characterization factors were those of the EF reference package 2 (EF 2.0) (EC-JRC et al., 2018; Fazio et al., 2018). Although the 16 EF impact categories were assessed (ESM 1), only 14 out of them (i.e., excluding ozone depletion (ODP) and ionizing radiation (IR)) were considered for the general results due to limitations of the Exiobase database (top-down approach). The 14 categories assessed were Climate change (CC), Human toxicity, non-cancer (HTOX_nc), Human toxicity, cancer (HTOX_c), Particulate matter (PM), Photochemical ozone formation (POF), Acidification (AC), Terrestrial Eutrophication (TEU), Freshwater Eutrophication (FEU), Marine Eutrophication (MEU), Land use (LU), Freshwater Ecotoxicity (ECOTOX), Water use (WU), Resource use, Fossils (FRD) and Resource use, Minerals and metals (MRD). For sensitivity purposes, the relevance of adopting the 16 EF categories is later discussed, compared to the limited use to 14 EF categories when employing the Exiobase database. Regarding HTOX_nc, the characterization factor for the zinc emissions from USEtox (Rosenbaum et al., 2008) was reduced to 2%, according to the procedure adopted in the IMPACT World + method (Bulle et al., 2019). Characterized values were normalized at the global level, referring to global impacts calculated by Crenna et al. (2019), and at EU-28 level for sensitivity purposes, by using the CF-TD results as reference. The EF2017 weighting factors (Sala et al., 2018) were applied to obtain a single score CF.

### 2.3 Geographical and temporal scope

The geographical and temporal scope depended on the assessment approach and component (domestic, trade, apparent consumption) (Table 2). Data availability at the country level defined the geographical scope, while data robustness determined the temporal scope. Calculations were performed for the period 2000–2014, however two main limitations arose:

- limitations of the top-down data collection: regarding top-down trade and consumption footprints, Exiobase data for 2000–2003 period showed multiple outliers in different impact categories while data were not available for the 2011–2014 period and were extrapolated (linear extrapolation excluding outlier years);
- limitations of process-based data collection: concerning the bottom-up approach (both trade and consumption footprints), calculations were made for four distinct years (2000, 2005, 2010, 2014). For the remaining years, the trade component was obtained based on interpolation according to the environmental results of the calculated total mass of imported and exported volumes (trade). The interpolated results were added to the calculated DF thereby obtaining an interpolated value for the CF-BU.

**Table 2:** Geographical and temporal scopes considered in this study, by approach and consumption component.

Approach	Consumption component	Geographical scope	Time frame
Top-down	Domestic	EU-28 Countries	2000–2014
	Trade Consumption	EU-28	2004–2014 (2011–2014 extrapolated)
		Countries	2004–2011
Bottom-up	Trade Consumption	EU-28	2000, 2005, 2010, 2014

Therefore, the criteria to define the timeframe 2005–2014 for this study were:

a) maximum inclusion of years available in both top-down (2004–2014) and bottom-up (2000, 2005, 2010, 2014) approaches to allow a comparative assessment;
b) maximum time coverage of more robust data available at EU level.

As the top-down approach is dependent on external data sources for the trade component (i.e., EXIOBASE), years beyond 2015 cannot be included in the assessment as extrapolation methods should be used, as already applied for the period 2012–2014. In this sense, enlarging the time period would include further uncertainties to the assessment.

### 2.4 Resource decoupling

The decoupling of the economic output from resource use due to EU consumption was assessed in order to outline the differences between resource and environmental decoupling. The Domestic Material Consumption (DMC) indicator was selected for this purpose, considering the following aspects. First, DMC has already been used in the decoupling literature (Table 1). Second, DMC is an indicator adopted within the EU Resource efficiency scoreboard (EC, 2016b). Third, although the Raw Material Consumption (RMC) quantifies the material footprint by considering the weight of materials extracted to produce them instead of the weight of the materials at the border (as in the DMC), the two indicators showed a similar trend along the time period evaluated (Eurostat, 2019) and resource decoupling results would be similar. When considering a territorial-based approach, the domestic extraction (DE) has been assessed.

### 2.5 Beyond the GDP: considering human well-being

Environmental decoupling has been mostly assessed considering the economic growth as reference (e.g., GDP), as indicated by the OECD (2002). However, some authors pointed out that overall sustainability, including further dimensions than the economic growth, should be considered (van den Bergh and Botzen, 2018). This is in line with the European Commission's “GDP and Beyond” Communication (EC, 2009b), where new indicators that include environmental and social aspects but remain as clear and appealing as GDP were promoted. van den Bergh (2009) discussed the GDP paradox, where GDP is a dominant and widely used economic indicator although being severely criticized for not properly representing social welfare in terms of development. In fact, GDP is an indicator that measures the economic activity rather than the well-being (Costanza et al., 2009). Thus, while the lack of comprehensivness of the indicator is agreed, GDP is still relevant and influences policy and economic decisions, which are mostly seeking the perpetuation of GDP growth (van den Bergh, 2009).

Alternatives to GDP were presented and evaluated in the literature (Costanza et al., 2009; van den Bergh, 2009). van den Bergh (2009) categorized them in four groups: (a) adjustments of GDP (e.g., Genuine Progress Indicator, GPI (Kubiszewski et al., 2013)); (b) sustainable or green versions of GDP (e.g., Sustainable National Income, SNI); (c) genuine savings (GS) (Bolt et al., 2002); and (d) composite indexes of relevant aspects of human well-being (e.g., Human Development Index, HDI). Among them, HDI is an alternative indicator with available annual data provided by the UN and worldwide coverage that can be employed in decoupling assessment. The employment of HDI as a human well-being indicator might be considered as a proper reference for evaluating decoupling, as already presented by UNEP (2011). Towards assessing the decoupling beyond GDP and considering human well-being, this study evaluated how HDI decouples from the environmental impacts of consumption.

## 3 Results

This section illustrates the evaluation of the decoupling related to the environmental impacts of EU consumption. In a first section, the decoupling assessment for the DF, CF-BU and CF-TD is reported. The resource decoupling is also assessed. Moreover, the environmental decoupling is analyzed at the country and impact category level. Finally, decoupling is assessed beyond GDP by employing the HDI indicator.

### 3.1 The decoupling of GDP from environmental impacts in the EU-28

**Fig. 3:**
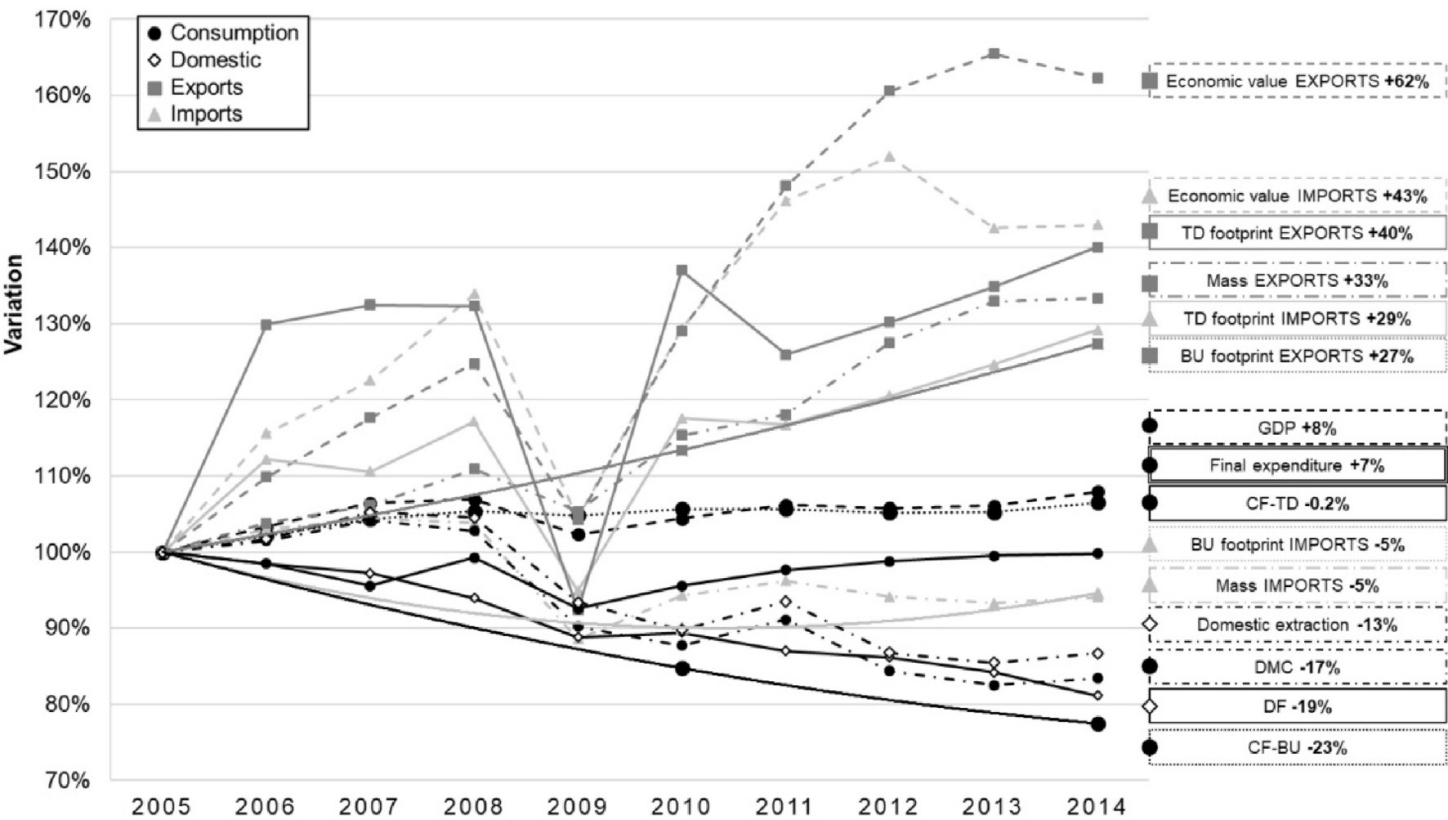
Production-, consumption-based and trade decoupling of EU-28 (2005–2014): Variation of the environmental impact indicators in relation with economic (economic value, GDP, final expenditure) and mass (mass, DMC, domestic extraction) trends.

The GDP showed decoupling from the environmental impacts of EU-28 consumption for the period 2005–2014 (Fig. 3). While the GDP grew by 8%, the single score CF had a variation between stable (−0.2%, CF-TD) to decreasing (−23%, CF-BU). Although the environmental impact decreased in both approaches (i.e., absolute decoupling), the CF-TD trend was close to stable (−0.2%) for this period, being in the limits of a relative decoupling. For all the indicators, the effect of the economic crisis can be observed in 2009, when the economic output (GDP = -4%) and the trade (imports = -22%, exports = -16%) decreased, in terms of single score. In general, the trade components had larger variations than the domestic one for the environmental impact indicators.

#### 3.1.1 Production-perspective: the decoupling of GDP from domestic environmental impacts in EU-28

During the assessed period (2005–2014), the single score DF was reduced by 19% following a continuous decrease. Compared to a GDP increase of 8%, the single score DF showed an absolute decoupling between 2005 and 2014. This result indicates the environmental impacts resulting from the consumption in EU-28 were reduced within the borders of the EU, i.e. considering a production- and territorial-based perspective, for the assessed impact categories.

#### 3.1.2 Consumption-perspective top-down: the decoupling of GDP from consumption-related environmental impacts in EU-28 (top-down approach)

The single score CF-TD showed a stable trend between 2005 and 2014 (−0.2%) (Fig. 3). Two different trends can be observed in this period: an irregular decrease between 2005 and 2009 (−7.5%) and a constant increase from 2009. This trend outlines the effects of the economic crisis started in 2008, where trade drastically decreased, as well as the potential increase of the environmental burdens of EU consumption if consumption patterns are kept beyond the assessed timeline (2014). Furthermore, the values from 2012 were extrapolated from the trends during the entire period, adding uncertainty to forecasting trends beyond the assessed period.

Assessing trade is crucial to understand the results since the environmental impact of DF showed a constant decrease during the evaluated period, contrarily to the increase of the environmental burdens of imports (+29%, single score) and exports (+40%, single score). In percentage, the exports had a larger increase than imports. However, the impacts of imports in the CF-TD were two times bigger than those of domestic and exports impacts, in absolute terms. Therefore, the increase of imports drastically affected CF-TD and compensated the decrease of domestic impacts and the increase of embodied environmental impacts exported from EU. Results in absolute terms are reported in the supplementary material (ESM 2).

#### 3.1.3 Consumption-perspective bottom-up: the decoupling of GDP from consumption-related environmental impacts in EU-28 (bottom-up approach)

The economic growth decoupled from the EU-28 CF-BU in absolute terms, as the single score indicator of the environmental impact decreased by 23% while the GDP grew by 8% (Fig. 3). As for the CF-TD, single score DF decreased by 19% and the environmental impacts of exports increased (27%, single score). However, the environmental burdens embodied in imports were also reduced (−5%, single score), in constrast to the results obtained in the top-down approach (i.e., where the impacts of imports increased). The trends of these components all resulted in a decrease in the total CF-BU. An “export effect” was observed, where EU-28 mainly imported food products and raw materials and exported finished products with complex supply chains. This implies a higher impact per unit of product. Contrary to the CF-TD, the DF was the most relevant component of the total environmental impact of CF-BU in absolute terms. Compared to the single score DF, the environmental burdens of imports represented from the 56% (2005) to the 64% (2014) and the exports between 27% and 42%. Results in absolute terms are reported in the supplementary material (ESM2).

#### 3.1.4 Resource decoupling

The resource use indicators domestic extraction (DE) and DMC were analyzed and both showed a decreasing trend between 2005 and 2014 (−13% and −17%, respectively) (Fig. 3). The behavior of these two indicators can be compared to the corresponding environmental impact indicators.

Both DE and DF decreased along the assessed period, however DF showed a higher decreased (−19%), outlining that the environmental decoupling was more intense than the resource decoupling. Although having a similar decrease between 2005 and 2014, the trend of both indicators varied along the period. While the DF followed a continuous decrease, the DE increased until 2007, drastically decreased between 2008 and 2010, as a result from the economic crisis, and presented an irregular decrease during the last period (2011–2014). In this sense, the DE had a relative decoupling until 2007 and an absolute decoupling for the rest of the period, apart from years of DE increase (e.g., 2011).

In the same line, CF-BU (−23%) showed an aligned behavior with the DMC (−17%) indicator as both decreased between 2005 and 2014, but this trend was more intense for the environmental impact than for the resource use. As well, while the CF-BU showed a decreasing trend along the entire period, the DMC increased during the first period (until 2009) and decreased from then.

The comparison of the pairs DE-DF and DMC-CF-BU unveiled a divergent trend between resource use and environmental impact indicators. This fact highlighted the contrast behavior of (a) single and composite indicators, and (b) resource depletion and environmental impact.

### 3.2 The environmental decoupling at country and impact category level

**Fig. 4:**
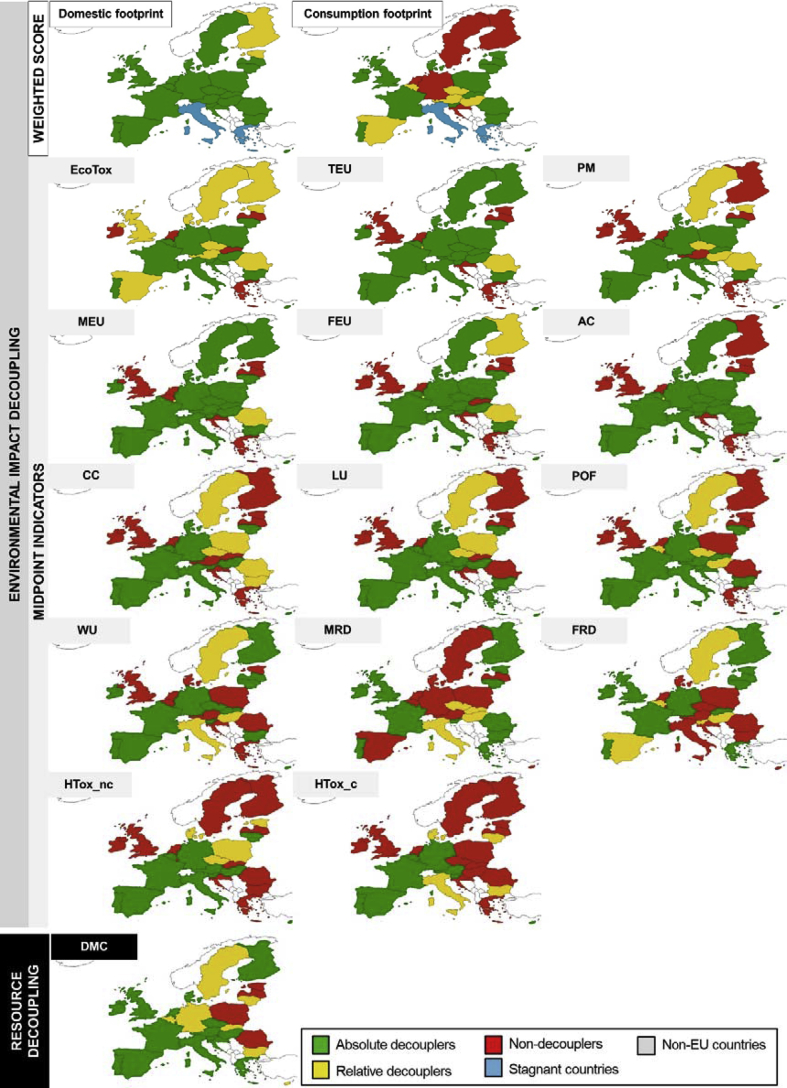
Decoupling at the country level (2004–2011): Environmental decoupling of the DF and CF-TD weighted scores, midpoint indicators of the CF-TD and Resources decoupling (DMC). Midpoint indicators are ordered by descending decoupling rate (ratio of number of decoupling countries and non-decouplers) (*Croatia refer to the period 2006–2011 due to data limitations).

The EU-28 as a single entity had a positive trend towards environmental decoupling. However, EU countries showed different and contrasting profiles. Fig. 4 shows the environmental decoupling of the EU-28 countries for the DF and CF-TD weighted scores between 2004 and 2011. While most of the countries showed an absolute decoupling for the DF, a distribution between non-decouplers, relative decouplers and absolute decouplers was found in the CF-TD. Trade is therefore the component driving the decoupling. Regarding the spatial distribution of the decoupling, this was less intense for northern countries (DF) and central countries (CF-TD). When comparing environmental decoupling and resource decoupling (i.e., relation between DMC and GDP variation in Fig. 4), resource decoupling obtained more positive values (number of absolute decouplers) than environmental decoupling.

#### 3.2.1 Consumption drivers for the different country patterns (2004–2011)

Examples of each decoupling group (i.e., absolute decouplers, relative decouplers, non-decouplers and stagnant) were evaluated to determine the drivers of the largest changes in the environmental impacts of individual countries. The consumption component, the flows, the economic sectors and the products leading the change in the environmental indicators were identified (ESM 3).

Absolute decouplers: Slovakia, Poland, Lithuania and Estonia. A decreased production of electricity from non-renewable sources (nuclear power, fossil fuels) (Lithuania), a variation in the domestic PM_2.5_ emissions (Estonia) and a decreased trade of manufactured products - particularly with electronic parts (e.g., vehicles, both in import and export) (Slovakia) and of bio-based products (e.g., wood, cereals, meat where the impact from import decreases while impact from export increases) (Lithuania, Estonia) motivated the decreasing trends in the CF-TD of absolute decouplers.

Relative decouplers: Czech Republic. Changes in CF-TD of relative decouplers were produced by increased food exports, reduced hard coal consumption compensated by increased crude oil imports, and enlarged domestic emissions of CO and CH_4_.

Stagnant countries: Greece and Italy. An improved energy mix and reduced food imports (Italy), improved wastewater treatment, decreased domestic SOx emissions and increased food imports (Greece) defined the impact trends of stagnant countries.

Non-decouplers: Luxemburg, Netherlands and Germany. An increased import of meat (Netherlands, Germany), increased import of metal manufactured products (Luxemburg) and increased imports of gold and other industrial minerals (Germany) were the main drivers towards a non-decoupling consumption.

#### 3.2.2 Environmental impacts driving decoupling and the effects of EU environmental policy

Regarding the midpoint indicators, Fig. 4 shows the impact categories sorted in descending order of number of decoupling countries (both relative and absolute). There were more decoupling than non-decoupling countries for all the impact categories. The leading impact categories towards decoupling were ECOTOX, TEU, PM and MEU with the highest amount of decoupling countries. Contrarily, HTOX categories had the lowest contribution towards a global EU-28 decoupling. Regarding absolute decoupling, TEU, AC and MEU emerged as the leading impact categories with between 19 and 18 countries as absolute decouplers. Geographically, south-west EU-28 area showed larger presence of decouplers for the different impact categories.

CC, PM and MRD had the main role, contributing to between 49% and 57% of the CF-TD weighted score during the entire period. FRD, WU, LU and AC represented more than 5% each for the entire period. While for CF-BU, CC (26–28%), PM (19–20%) and FRD (18–19%) were the most contributing categories to the weighted score for years 2005 and 2010 (ESM 4). The relevance of these categories in the weighted score was also related to the weighting factors adopted in the EF2017 scheme (Sala et al., 2018) (ESM 1).

During the assessed period, the impact categories showed a decreasing trend (up to −30%, AC) for the CF-TD approach, apart from HTOX_c (+1%) and MRD (+127%). In fact, MRD was the only category that showed no decoupling from the GDP, which is in contrast with the reduction observed for the pressure indicator DMC. On the contrary, all the indicators followed an absolute decoupling trend for the CF-BU, ranging between −1% (LU) and −88% (HTOX_nc) (ESM 5). Furthermore, HTOX_c obtained negative values in the CF-BU, indicating that the embodied impacts in exports were higher than the domestic and imported ones. This is a clear limitation of the representative products currently modelled. Hence, results are illustrative of the possible methdological approach but are still not mature to be used to draw conclusions on the impacts trend.

**Fig. 5:**
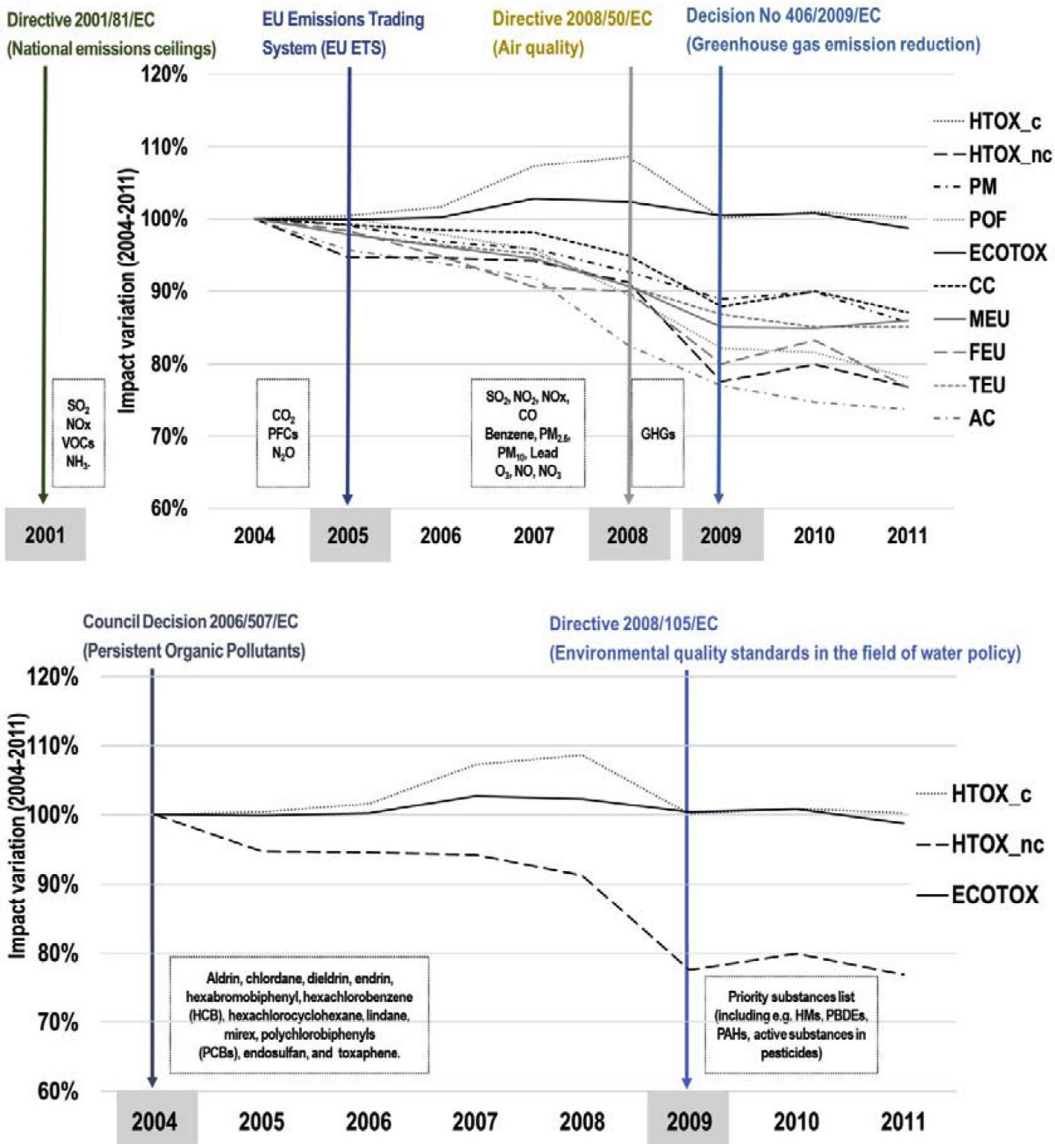
Evolution of DF impact categories and entry into force of related EU environmental policies.

Furthermore, the analysis of DF at the indicator level allows for assessing the possible effects of EU policies acting on the environmental aspects. The effect in the environmental burdens of some policies that entered into force within the considered timeframe or a few years before (EC, 2009a, 2008a; 2008b, 2006, 2003, 2001) was observed (Fig. 5). The foreseen reduction of certain types of emissions or a ban of a number of chemicals (e.g. active substances in pesticides) mainly contributed to a reduction in the impact, even if sometimes the reduction was not very significant. Along the assessed period, the effect of the global economic crisis of 2008 should be considered, which contributed to the decreasing trend. Most of the impact categories with flows associated to specific policies showed the highest decrease between 2008 and 2009 due to reduced trade and consumption levels. Among the impact categories, ecotoxicity was the one showing the lowest effect due to the economic crisis, contrarily to the human toxicity – cancer that had the largest effect.

#### 3.2.3 Assessing decoupling beyond GDP

**Fig. 6:**
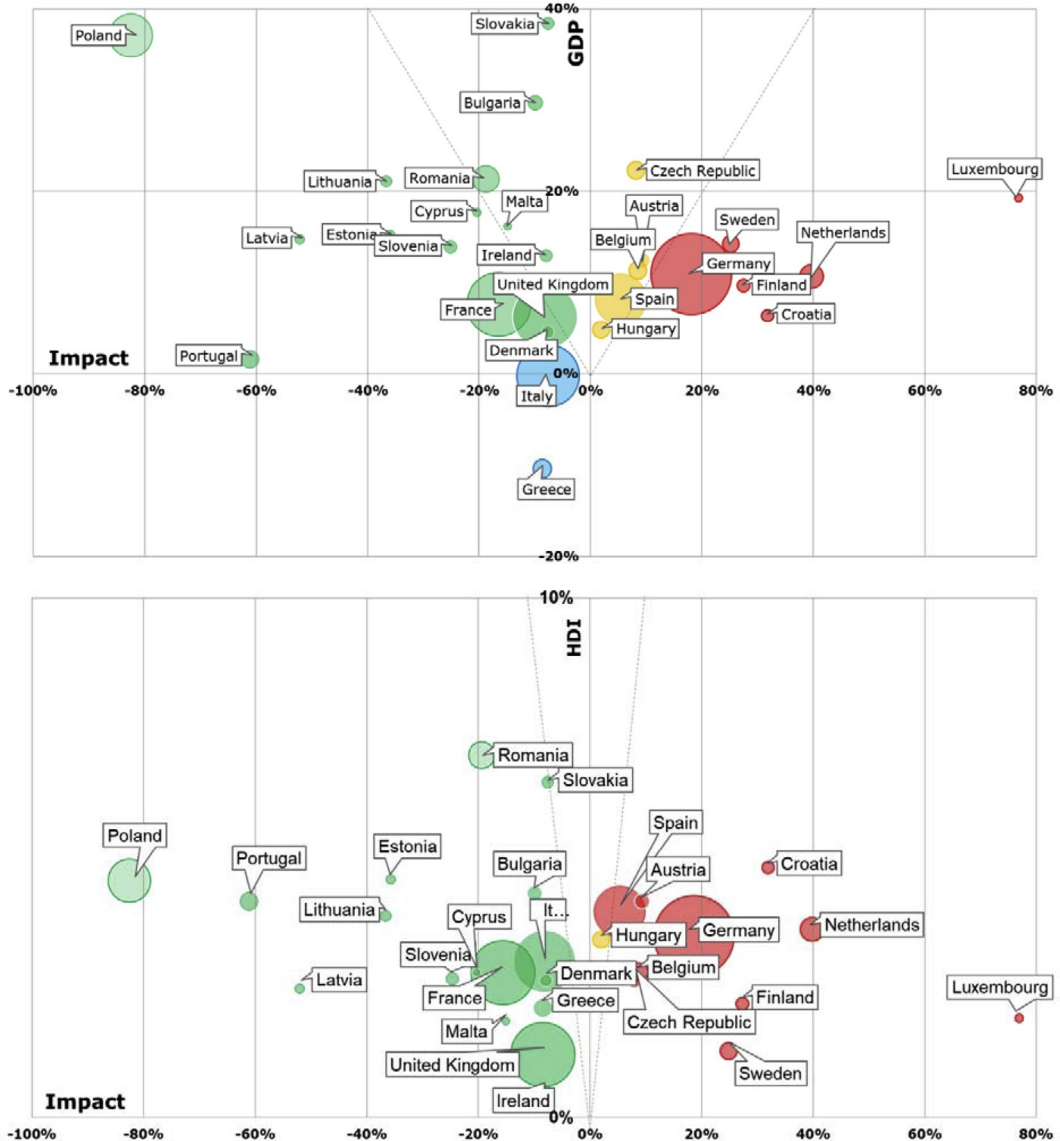
Decoupling at the country level (2004–2011): Decoupling the economic output (GDP) and the human well-being (HDI) from the CF-TD weighted score. Bullet size represents country population (2010). Countries are classified in absolute decouplers (green), relative decouplers (yellow), non-decouplers (red) and stagnants (blue). (For interpretation of the references to colour in this figure legend, the reader is referred to the Web version of this article.)

Concerning the assessment of whether human well-being decoupled from the environmental impacts of consumption and how the result varies from the GDP decoupling, Fig. 6 compares the environmental decoupling of the economic output (in terms of GDP) and the human well-being (in terms of HDI) from the CF-TD.

When comparing the use of the two reference indicators (GDP and HDI), some trends were observed. In general, the environmental decoupling employing HDI was more dichotomic than when using GDP and countries were absolute decouplers or non-decouplers, apart from Hungary that showed a relative decoupling in both cases (i.e., the CF-TG growth of 2.1% was lower than the increase of both indexes: 5% - GDP - and 3.5% - HDI). The number of countries which are non-decouplers and absolute decouplers increased compared to the graph illustrating decoupling to GDP. Firstly, those countries with a decreasing environmental impact trend (between −7 and −80%) were absolute decouplers considering both the GDP and the HDI. Furthermore, the group of stagnant countries (Italy and Greece) disappeared and became absolute decouplers because, contrary to the GDP, the HDI increased for all the EU-28 countries along the assessed period. Secondly, as GDP had growing rates larger than the HDI, the non-decouplers showed the same behavior for GDP and HDI as the environmental impact growth was still higher (between 17 and 76%). Finally, most of the countries with relative decoupling when using GDP had a larger CF-TD growth (5.5–9.4%) than the HDI variation (2.9–4.2%), resulting in non-decouplers when assessing well-being. In this last case, the HDI increase was between 1.4 and 8 times smaller than the GDP one.

In terms of population, four out of the six most populated EU countries (i.e, France, Italy, United Kingdom, and Poland) were in both cases either decouplers or stagnant (Italy in the decoupling assessment with the GDP indicator). While Germany showed no environmental decoupling in both cases, the GDP of Spain had a relative decoupling from the environmental impacts but the HDI showed no decoupling. Similarly to Spain, some of the mid-populated countries (10–20 million inhabitants) changed from relative decouplers (GDP) to non-decouplers (HDI) (i.e., Belgium, Czech Republic), apart from Hungary which remained as relative decoupler in both cases. The rest of mid-populated countries revealed as specific cases: The Netherlands was a non-decoupler in both cases, Portugal was an absolute decoupler for both indicators and Greece changed from stagnant (GDP) to absolute decoupler (HDI). The least populated countries (<3 million inhabitants) showed absolute decoupling in both cases, apart from Luxembourg which resulted in non-decoupler (both GDP and HDI). In absolute terms, assessing the GDP decoupling from the environmental impacts of consumption identified a larger amount of population as decoupler or being stagnant (383 million inhabitants) than when considering HDI (353 million inhabitants). Such trend implied that for each inhabitant having a consumption pattern without decoupling, 3.25 inhabitants were having consumption patterns that showed decoupling when using the GDP as economic indicator and only 2.4 when considering the well-being indicator HDI.

## 4 Discussion

### 4.1 The environmental decoupling in the EU-28

The environmental impacts of EU-28 consumption showed decoupling during the last decades (2005–2014) as suggested by the literature for waste generation (Mazzanti and Zoboli, 2008) and for carbon footprint (Liobikiene and Dagiliute, 2016), as well as for the Ecological Footprint trends (Lin et al., 2018). These results are aligned with global assessments (Schandl et al., 2016; Wood et al., 2018), which indicated that relative decoupling takes place for some environmental issues while global absolute decoupling is only possible for some aspects (e.g. land use, Wood et al., 2018). However, country-level results varied from the literature. For example, Liobikiene and Dagiliute (2016) found that Croatia, Italy, Portugal and Spain were non-decouplers regarding climate change for the period 1993–2010, and only three countries were performing as absolute decouplers (Denmark, Estonia, Germany). In this assessment, while Croatia and Italy were non-decouplers for climate change, half of the countries (14) were behaving as absolute decouplers for 2005–2014.

The assessment stressed the relevance of consumption-based approaches in assessing the decoupling phenomenon as discussed in the literature (Hamilton et al., 2018; Wiedmann and Lenzen, 2018; Wood et al., 2018). DF decoupling was more intense than CF decoupling and, thus, the economic growth showed a higher level of decoupling from the environmental impacts in the territory than from those impacts taking place abroad. Therefore, EU-28 was a net importer of embedded environmental impacts in traded goods. Hence, environmental policies should address also trade products to integrate non-domestic environmental burdens. In the same line, the necessity to evaluate the decoupling from environmental impacts rather than resource use (UNEP, 2011) was also demonstrated. Although DF and DE as well as CF and DMC showed the same trend, the behavior of resource depletion varied from the environmental impact one. Furthermore, the study highlighted the differing results when employing individual impact indicators and a single weighted score.

### 4.2 Modeling the environmental impacts of consumption

#### 4.2.1 Environmental modeling approaches

Input-output LCA and process-based LCA were contrasted by confronting CF-TD and CF-BU, respectively. In both approaches, the CF was higher than the DF and, thus, EU-28 was a net importer of environmental impacts. One of the main divergences between both approaches relied on the MRD impact category. MRD represented only 2% of the impact in CF-BU but was a major driver in CF-TD (from 13% to 24%) as the inventory was highly aggregated in Exiobase and different types of minerals were characterized with a singular characterisation factor, as discussed in Beylot et al., 2019.

The following differences between the two environmental modeling approaches arose as limitations of this comparison:

- A DF based on statistical data was merged with a trade footprint calculated based on two different modeling approaches: process-based LCA (CF-BU) and input-output-based LCA (CF-TD).
- The elementary flows coverage was not the same for these components in the calculation of the environmental impacts of consumption: around 2,000 (DF), around 15,000 (process-based LCA trade footprint in CF-BU) and 78 (input-output-based LCA trade footprint in CF-TD).
- The top-down approach (CF-TD) with an input-output-based LCA trade footprint included the impacts of matter-less economic sectors (e.g., services), while these were excluded from the bottom-up approach (process-based LCA).
- The top-down approach (CF-TD) was more correlated to monetary flows while the bottom-up (CF-BU) one followed the trend of mass flows. Regarding CF-BU, the decrease of the environmental impact can be related to the fact that more semi-finished materials or finished products are consumed in the EU. This could be the real issue at stake, i.e., the material footprint embedded is higher but the mass which is imported is less. This again my question the represenativness of the selected products.

Therefore, from an inventory perspective, a compromised solution combining the benefits of both approaches might provide with results better mirroring the reality.

#### 4.2.2 Influence of life cycle assessment inventory modeling and normalization

The normalization and weighting of the characterised values and the inclusion of different midpoint impact categories can alter the single footprint outputs and the decoupling assessment. While previous literature showed no normalization steps and used single indicators, the sensitivity analysis of varying these aspects in LCA unveiled a considerable influence. The evaluated results followed a global normalization step to show the evolution of EU-28 environmental impacts compared to global environmental impacts, and included 14 EF categories. However, the assessment at the country level can be also performed applying EU-28 normalization factors to compare the countries with an EU reference. All the 16 EF categories can be assessed for DF (Table 3) (ESM 6).

**Table 3:**
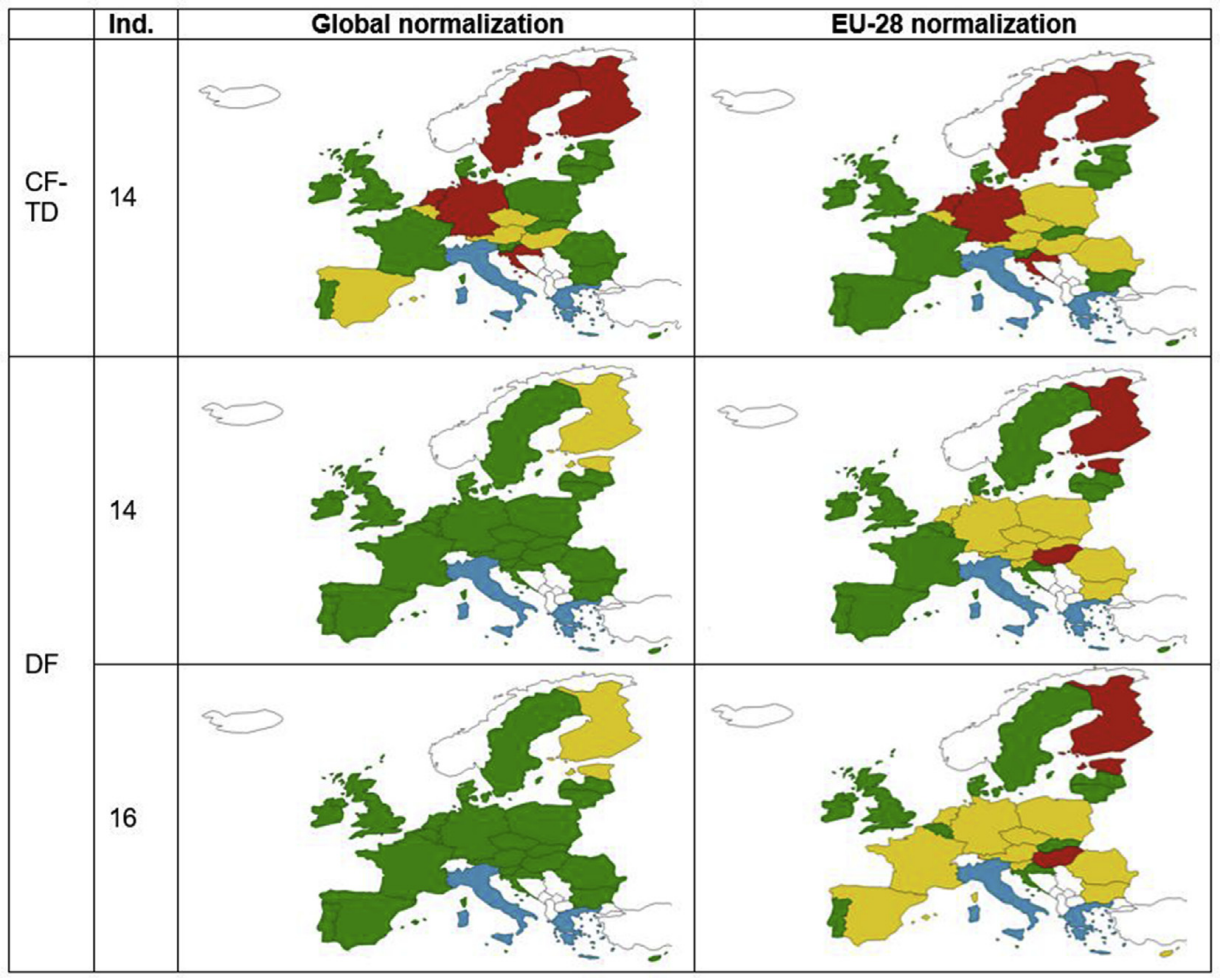
Decoupling assessment at the country level of the CF-TD and DF with different normalization and number of impact categories (2004–2011). Green: asbolute decoupling; yellow: relative decoupling; red: non decoupling; blue: stagnant.

The sensitivity assessment unveiled some divergences. First, global normalized results were more positive than EU-28 ones as the EU-28 share of the global environmental impacts was limited (<10%). Thus, while globally normalized DF results showed an almost-green map, absolute decouplers were more restricted in EU-normalized ones. Second, using EU-28 normalization sets the average EU-28 trend for a determined timeframe (i.e., average of the variations in the different EU countries) as the reference behavior, leading to potential opposite trends in the characterized and the normalized values. For example, although the AC impact of all EU-28 countries decreased following the policy implementation against related emissions, Luxemburg's decreasing rate (−4%) was lower than the EU average, resulting in an increasing normalized trend (+28%).

Third, the role of each impact category varied regarding the normalization type as the global share of EU-28 impacts depends on the midpoint category: EU-28 had more relevance in IR (68%), PM (12%), CC (9%) and FEU (8%), while a low effect to LU (3%) and MRD (2%) (ESM 6). Thus, the contribution of each country to the midpoint categories can result in a different decoupling output based on the normalization and the number of impact categories included in the assessment (Table 3). In particular, some countries showed a higher sensitivity to specific categories. For example, France was an absolute decoupler in all cases, apart from DF with global normalization and 16 indicators. Finally, IR and ODP had a relatively high weighting score compared to other impact categories in the EF weighting set, so they affected the single weighted score. See (ESM1) for the list of the EF weighting factors.

#### 4.2.3 Temporal scope

Defining the temporal scope of a decoupling assessment can influence the results due to specific events (e.g., economic crises) Although the CF-TD showed a stable trend for 2005–2014, the annual decoupling assessment unveiled that decoupling occurred at the beginning (2005–2007) and ending (2012, 2014) years while non-decoupling took place along the period 2008–2011, due to the economic crisis and the decreased economic output, and 2013 (ESM 8). Regarding the midpoint categories, none of them followed the same pattern along the entire timeframe and the economic crisis severely affected 2009 and 2010. The categories that showed more years of absolute decoupling were FEU, CC and LU, antagonistically to HTOX_c. The assessment of the annual decoupling is common in literature (Chen et al., 2017; de Freitas and Kaneko, 2011; Wang and Yang, 2015; Zhang and Wang, 2013) and can show the effects of environmental policy programs and their implementation. However, annual assessments must be complemented with complete timeframe observations to understand the behavior of an economy as annual decoupling can be offset with non-decoupling behaviors in the following years (and vice versa).

Regarding DF, robust data was available for the period 2000–2014 (ESM 9), which showed slightly more absolute decoupling in EU-28. Italy and Greece were stagnant in both cases as GDP was stable (0%) or decreasing (recession). While both Finland and Estonia were relative decouplers for 2005–2014 due to an increase of MEU and MRD (Finland), and CC and HTOX_c (Estonia), only Latvia was a relative decoupler for the longer period (2000–2014) due to an increase in CC. Decoupling assessment of longer periods could provide information about the mid- and long-term effects of policy implementation. In this case, EU-28 is leading the development and implementation of environmental policies as observed in the absolute decoupling results for the DF, which were even better for a longer evaluation timeframe.

#### 4.2.4 Environmental impacts and decoupling: uncertainties, limitations and missing aspects

The assessed environmental indicators (DF, CF-TD and CF-BU) encompassed multiple environmental pressures and impacts (at least 14 categories), thereby attempting a comprehensive accounting of the environmental impact of EU-28 consumption. This represents a clear novelty of this work compared to previous studies. In the literature, most of the analysis focused on a single environmental impact and the environmental modeling was very limited. For instance, some studies assessing climate change only accounted for CO_2_ emissions with results indicating a very limited relative decoupling and, thus, the consideration of all GHG emissions could result in a non-decoupling behaviour (e.g. Zhang and Wang, 2013).

The extensive impact assessment conducted in this study was highly data demanding, and it relied on a wide variety of sources and models to populate the underpinning environmental pressures. For calculating the inventory of emissions and resources, data were retrieved from: public statistics, environmental modelling for data gap filling, life cycle inventories, multi-regional input-output databases, as detailed in Sala et al., (2019a). The complexity of the exercise (bringing together more than 3000 type of emissions and resources, over 15 years and for 28 EU Member States) and the use of models imply a degree of uncertainty in the results, which is difficult to estimate at this stage. This should be kept in mind while interpreting the results.

Besides, there is an increasing awareness of the level of impacts posed by current patterns of production and consumption, e.g. leading to an unprecedented decrease in biodiversity at global level (Johnson et al., 2017) and in biotic stock (e.g. fish declining) (Vasilakopoulos et al., 2014). This suggests that the current assessment of decoupling, despite covering 14 impact categories, should be further complemented in order to be able to better capture current trends in environmental impacts. In fact, although some attempts have been made to integrate missing aspects into the LCA framework, some environmental impacts related to consumption are not included in current LCA practice; e.g., noise (Cucurachi, 2014), marine litter, light contamination and some drivers of biodiversity loss (such as biotic resource overexploitation or detrimental effects of invasive species (Hanafiah et al., 2011)). Moreover, even absolute decouplers might generate impacts which are far beyond the planetary boundaries (Sala et al., 2019a) and this supports the need of complementing, as well as with the evaluation of additional impacts currently missing. The paradigm of resource efficiency and environmental efficiency, underpinning the decoupling assessment, is not enough to assess the sustainability of production and consumption systems.

## 5 Conclusions

This paper evaluates the decoupling of GDP from the environmental impacts of EU-28 consumption by employing LCA-based environmental indicators and exploring the methodological aspects that influence the decoupling assessment and resulting outputs. The methodological approach and results contribute to the literature by adopting an LCA perspective that assesses environmental decoupling in impact terms rather than pressures, as well as using a set of multiple impact categories.

The environmental impacts of EU-28 consumption showed decoupling during the last decades (2005–2014), behaving as between relative (−0.2% change) and absolute (−23%) decoupler, depending on the environmental modeling approach taken. However, decoupling had a different intensity along the EU territory. Some countries showed higher decoupling levels compared to others, thereby displaying a heterogeneous map of the decoupling in the EU-28. In general, southern countries showed better results (i.e., higher amount of absolute decouplers) than northern countries, apart from water consumption.

Trade variation and the economic crisis of started in 2008 had a strong effect on the results. The CF was higher than the DF highlighting the role of EU as a net importer of environmental burdens. The imports of meat, minerals for manufacturing products, manufactured products, and non-renewable energy sources were the main contributors to increased environmental burdens in non-decoupling countries. The economic crisis severely affected trade patterns and the environmental burdens decreased during the following two years due to a lower consumption in EU countries. This positively affected the decreasing trend for the assessed period. Towards exploring the economic changes behind the trend of the environmental impacts, a decomposition analysis could shed light on whether structural changes, technology progress or trade patterns were the main contributors (e.g., Koh et al., 2016).

The decoupling of GDP from environmental impacts of EU consumption was led by acidification, particulate matter, land use, and eutrophication impacts. The results stressed out the need to assess decoupling from a consumption perspective towards including the environmental burdens taking place in developing countries where environmental policy is less strict. Accordingly, environmental policy could force changes beyond the EU-28 territory by considering traded products. This would broaden the current positive effect in the EU territory, according to the assessment of implemented EU policies and the trend of the affected environmental impact categories.

Methodologically, the top-down approach showed higher values of environmental impact in absolute terms and a stable trend along the assessed period. Input-output-based LCA considers all the economic activities, including the immaterial ones (i.e., services). However, the quantification of the environmental burdens is less accurate than in process-based LCA due to the constraints of the environmental extensions. On the contrary, the bottom-up approach showed better results and trade had larger relevance than domestic impacts. Process-based LCA excludes the services of the environmental modeling and it is based on representative products. Since both approaches show drawbacks, a more accurate accounting of the environmental decoupling might be situated between the top-down and the bottom-up approaches.

Furthermore, the way of modeling the environmental impacts was key in the assessment of decoupling and further improvements might be performed to better mirror the reality. In the same line, data availability and origin influenced the robustness of the calculations as different completeness of data added uncertainty to the results. On the other hand, the paper discussed how life cycle modeling and impact assessment can affect the results by observing the sensitivity to different normalization (global, EU-28), number of indicators (14, 16) and timeframe. EU-28 role in global environmental impacts as well as the weighting scheme of EF2017 were revealed as key aspects in the total outputs. As well, the impact assessment method employed (EF2017) missed some environmental impacts related to consumption (noise, marine litter, light contamination and some drivers of biodiversity loss). Further research might address the modeling of these missing environmental aspects related to consumption in life cycle assessment.

Following discussions about the relevance of GDP but the constraint vision of this economic indicator to track progress, the study employs HDI to evaluate the decoupling of human well-being from environmental impacts. Assessing decoupling beyond GDP allows exploring the environmental decoupling of human well-being by considering social sustainability aspects rather than exclusively economic development. While the HDI can be a useful indicator for this purpose with available annual worldwide data, the Genuine Progress Indicator (GPI) (Kubiszewski et al., 2013) would be an interesting metric when data are worldwide available in order to integrate further elements of sustainability in the consideration of human well-being.

However, to assess sustainability of production and consumption, the decoupling assessment is not enough. Understanding to which extent the impacts transgress planetary boundaries it is essential to ensure that the decoupling is not only a measure of eco-efficiency but an effective indication of an improved sustainability.
