# Supplementary material for: Assessing the decoupling of economic growth from environmental impacts in the European Union: A consumption-based approach
Source: J Clean Prod. 2019 Nov 1;236:117535. doi: 10.1016/j.jclepro.2019.07.010 (PMC6737992; doi:10.1016/j.jclepro.2019.07.010)
Supplement: Multimedia component 1 [file mmc1.docx]

**Electronic Supplementary Material**

**Assessing the decoupling of economic growth from environmental impacts in the European Union: a consumption-based approach**

Sanyé-Mengual E^1^, Secchi M^1^, Corrado S^1^, Beylot A^1^, Sala S^1,^*.

^1^ European Commission, Joint Research Centre, Via Enrico Fermi 2749, I-21027 Ispra, Italy

*Corresponding author: serenella.sala@ec.europa.eu

**Index**

[ESM 1. Impact categories of the environmental footprint (EF2017 method) 2](#_Toc13494637)

[ESM 2. Absolute values of DF, CF-TD and CF-BU by consumption component 3](#_Toc13494638)

[ESM 3. Drivers of Consumption Footprint top-down decoupling at the country level: group examples (global-normalised). 4](#_Toc13494639)

[ESM 4. Contribution of midpoint categories to weighted score (2004-2011). 10](#_Toc13494640)

[ESM 5. Trends of midpoint categories (2005-2014). 11](#_Toc13494641)

[ESM 6. Life cycle parameters: Decoupling assessment at the country level (EU-28 normalisation). 13](#_Toc13494642)

[ESM 7. Drivers of Consumption Footprint top-down decoupling at the country level: group examples (EU-28-normalised). 15](#_Toc13494643)

[ESM 8. Temporal scope: Annual decoupling 18](#_Toc13494644)

[ESM 9. Temporal scope: Domestic footprint decoupling (2000-2014; 2005-2014, 2004-2011) 19](#_Toc13494645)

[References 20](#_Toc13494646)

# ESM 1. Impact categories of the environmental footprint (EF2017 method)

The 16 impact categories considered in the EF2017 method (EC, 2017) are detailed in table 1.A.

**Table 1.A.** Impact category, acronym, unit and Weighting factors (WFef) for the Environmental Footprint (EF) context.

| **Impact category** | **Abbreviation** | **Unit** | **WFef** |
| --- | --- | --- | --- |
| Climate change | CC | kg CO_2_ eq | 21.06 |
| Ozone depletion | ODP | kg CFC-11 eq | 6.31 |
| Human toxicity, non-cancer | HTOX_nc | CTUh | 1.84 |
| Human toxicity, cancer | HTOX_c | CTUh | 2.13 |
| Particulate matter | PM | Disease incidence | 8.96 |
| Ionizing radiation, human health | IR | kBq U^235^ eq | 5.01 |
| Photochemical ozone formation,  human health | POF | kg NMVOC eq | 4.78 |
| Acidification | AC | molc H^+^ eq | 6.20 |
| Eutrophication, terrestrial | TEU | molc N eq | 3.71 |
| Eutrophication, freshwater | FEU | kg P eq | 2.80 |
| Eutrophication, marine | MEU | kg N eq | 2.96 |
| Ecotoxicity, freshwater | ECOTOX | CTUe | 1.92 |
| Land use | LU | Pt (points) | 7.94 |
| Water use | WU | m^3^ world eq | 8.51 |
| Resource use, fossils | FRD | MJ | 8.32 |
| Resource use, mineral and metals | MRD | kg Sb eq | 7.55 |

# ESM 2. Absolute values of DF, CF-TD and CF-BU by consumption component

The following table displays the absolute values of the Domestic Footprint (DF), the trade footprints of the top-down approach (import-TD, export-TD), the consumption footprint top-down (CF-TD), the trade footprints of the bottom-up approach (import-BU, export-BU), and the consumption footprint bottom-up (CF-BU). The absolute values allows for observing the relevance of the different consumption components in the two modeling approaches taken: input-output-based LCA (TD) and process-based LCA (BU).

**Table 2.A.** Comparison of decoupling results for the domestic footprint (14 indicators, global normalized) for two different time periods: 2005-2014 and 2000-2014.

| **Impact category** | **Unit** | **DF** | **Import TD** | **Export TD** | **CF-TD** | **Import-BU** | **Export-BU** | **CF-BU** |
| --- | --- | --- | --- | --- | --- | --- | --- | --- |
| Human toxicity,  cancer | CTUh | 1.23E+04 | 3.97E+04 | 2.58E+04 | 2.62E+04 | 2.94E+04 | 6.72E+04 | -2.55E+04 |
| Human toxicity,  non-cancer | CTUh | 1.02E+05 | 1.26E+06 | 1.17E+06 | 1.92E+05 | 2.23E+05 | 2.89E+05 | 3.62E+04 |
| Particulate matter | disease  incidences | 4.97E+05 | 6.23E+05 | 2.97E+05 | 8.24E+05 | 2.42E+05 | 9.06E+04 | 6.48E+05 |
| Photochemical  ozone formation | kg NMVOC eq | 1.59E+10 | 1.95E+10 | 1.12E+10 | 2.42E+10 | 7.93E+09 | 2.26E+09 | 2.16E+10 |
| Ionising radiation | kBq U^235^ eq | 6.01E+11 | N/A | N/A | 6.01E+11 | 4.82E+10 | 4.35E+10 | 6.06E+11 |
| Water use | m^3^ eq | 3.07E+12 | 3.45E+12 | 9.18E+11 | 5.60E+12 | 9.58E+11 | 7.56E+11 | 3.27E+12 |
| Ecotoxicity  freshwater | CTUe | 2.73E+12 | 1.20E+12 | 1.01E+12 | 2.92E+12 | 3.04E+12 | 1.90E+12 | 3.89E+12 |
| Climate change | kg CO_2_ eq | 4.82E+12 | 3.67E+12 | 1.91E+12 | 6.59E+12 | 9.59E+11 | 6.91E+11 | 5.09E+12 |
| Resource use,  fossils | MJ | 2.71E+13 | 8.47E+13 | 2.95E+13 | 8.23E+13 | 5.58E+13 | 1.49E+13 | 6.78E+13 |
| Ozone depletion | kg CFC-11 eq | 9.18E+06 | N/A | N/A | 9.18E+06 | 8.26E+05 | 2.82E+04 | 9.97E+06 |
| Eutrophication,  marine | kg N eq | 8.56E+09 | 6.82E+09 | 4.29E+09 | 1.11E+10 | 2.45E+09 | 1.00E+09 | 1.00E+10 |
| Eutrophication  freshwater | kg P eq | 5.22E+08 | 2.09E+08 | 6.09E+07 | 6.71E+08 | 7.96E+07 | 9.43E+07 | 5.08E+08 |
| Land Use | pt | 7.48E+14 | 7.62E+14 | 2.00E+14 | 1.31E+15 | 3.21E+13 | 3.47E+13 | 7.45E+14 |
| Eutrophication,  terrestrial | mol N eq | 9.15E+10 | 1.09E+11 | 5.33E+10 | 1.47E+11 | 2.19E+10 | 7.10E+09 | 1.06E+11 |
| Acidification | mol H^+^ eq | 2.45E+10 | 3.93E+10 | 1.93E+10 | 4.44E+10 | 1.79E+10 | 4.02E+09 | 3.84E+10 |
| Resource use,  minerals and metals | kg Sb eq | 8.21E+06 | 2.48E+08 | 1.32E+08 | 1.24E+08 | 3.71E+06 | 6.41E+06 | 5.51E+06 |

# ESM 3. Drivers of Consumption Footprint top-down decoupling at the country level: group examples (global-normalised).

**Table 3.A.** Explanation of examples of different behaviour in the Consumption Footprint top-down decoupling (2004-2011) for 14 indicators, by country: category variation (weighted and characterized value), consumption element and explanation.

|  | **Country** | **Category** | | **Consumption**  **element** | **Explanation** |
| --- | --- | --- | --- | --- | --- |
|  |  | **Weighted** | **Characterised** |  |  |
| Non- decouplers | LU | HTOX_c:  +431% | +431% | Import | Chromium to air emission from manufacturing sectors (especially linked to metals and equipments imports) increase (5x) during the considered timeframe. |
|  |  | MRD:  +192% | +192% | Import | Impact due to imports increased in the considered timeframe. This is mainly due to the increase of quarrying activities of sand and clay imported (generating the impact related to “other industrial minerals”). |
|  |  | ECOTOX:  +52% | +52% | Import | Chromium to air emission from manufacturing sectors (especially linked to metals and equipments’ imports) increase (5x) during the considered timeframe. |
|  |  | CC:  +32% | +32% | Import | Imports of Manufacture of rubber and plastic products (25) increase, causing an increase in CH4 and CO2 emissions. |
|  |  | FRD:  -381% | -381% | Export | The impact of exports increase (x4) along the assessed period. In 2004, as both trade elements have a negative impact. Data from Exiobase indicate there are negative flows of natural gas due to the economic sectors Production of electricity by biomass and waste, Manufacture of motor vehicles, trailers and semi-trailers (34), and Manufacture of rubber and plastic products (25). |
|  |  | HTOX_nc:  -194% | -194% | Import | The environmental burdens of imports decrease during the assessed period.  There are negative emissions of Arsenic in 2004 due to import of Production of electricity by biomass and waste (Exiobase data). |
|  | NL | FRD:  +193% | +193% | Import | Increase in the import impact due to a larger import of natural gas: extraction activities of natural gas (WM.), manufacturing of iron and steel, manufacturing of plastics and rubber. |
|  |  | TEU:  +86% | +86% | Import | Increase in imported ammonia emissions to air due to imports of meat (cattle, pigs and poultry) (WL, GB, DE, WM). |
|  |  | FEU:  +67% | +67% | Import | Increase in the emission of phosphorus (to soil and to water). This is due to the intensification in the import of processing activities of meat cattle and to the production of wearing apparent. |
|  |  | MEU:  +76% | +76% | Import | Increase in imports and the embodied N emissions to water due to meat pigs (DE), meat cattle (GB), cereals (FR) and meat poultry (WM). |
|  |  | HTOX_c:  +63% | +63% | Import | The increase of imported manufacture of iron and steel (in particular from WE and RU) generated an increase in the emission of chromium to air (+98%). |
|  |  | HTOX_nc:  +60% | +60% | Trade | The increase of imported manufacture of iron and steel (in particular from WE and RU) generated an increase in the emission of lead to air (+122%).  Moreover, the impact of lead to air (+141%) derived from the extraction of exported natural gas increases the total impact of Export.  Despite that, the total impact due to Import is always higher (1.5 times) than the increase in total Export, leading to an intensification of the impact in this category of the Consumption Footprint. |
|  |  | AC:  +50% | +50% | Trade | Increase in imports and decrease in exports, leading to a higher contribution of ammonia emissions to air, mainly due to imports of meat (cattle, pigs and poultry) (WL, GB, DE, WM). |
|  | DE | MRD:  +38% | +38% | Import | Increase in imports’ impact due to increase of the depletion of gold [Chemicals nec (WL), Mining of precious metal ores and concentrates (MX) and Precious metals production (RU)] and other industrial minerals [Construction (45) and Chemicals nec from China]. |
|  |  | FRD:  --135% | -135% | Trade | Decrease in exports (-57%) due to uranium, and increase in imports (+48%). Regarding imports, even crude oil has decreased in this period, there is an increase in the impact related to natural gas as this was negative in 2004 (manufactured products: Manufacture of motor vehicles, trailers and semi-trailers (34), Manufacture of rubber and plastic products (25), Manufacture of office machinery and computers (30), Manufacture of furniture; manufacturing n.e.c. (36). Current natural gas is imported from Middle East and Norway. |
| Relative | CZ | HTOX_c:  +56 | +56% | Trade | Decrease of Export and increase of Import play the main role in this result. Chromium to air emission due to import of manufactured steel basic iron and steel and of ferro-alloys increases in the timeframe considered. |
|  |  | FRD:  +37% | +37% | Trade | Natural gas amount (and impact) in 2004 is negative both for Import and Export (mainly due to Manufacture of rubber and plastic products 25).  Then, in the following years, the impact linked to natural gas increase. In 2011, for example, the import of plastic products is the top contributor to the impact. |
|  |  | MEU:  -20% | -20% | Domestic | The emission of NOx to air decreases in the Domestic component. This reduction is mainly linked to the public power and Industry sectors. |
|  |  | TEU: -20% | -20% | Domestic | The emission of NOx to air decreases in the Domestic component. This reduction is mainly linked to the public power and Industry sectors. |
| Absolute | SK | HTOX_c:  -66% | -66% | Trade | Decrease of imports and exports, declining chromium emissions from manufactured products (e.g. medical, precision and optical instruments, watches and clocks, radio, television and communication equipment and apparatus, furniture, motor vehicles, machinery and equipment). |
|  |  | HTOX_nc:  -57% | -57% | Trade | Decrease of imports and exports, reducing the emissions of lead and mercury from manufactured products (e.g. medical, precision and optical instruments, watches and clocks, radio, television and communication equipment and apparatus, furniture, motor vehicles, machinery and equipment). |
|  |  | FRD:  +15% | +15% | Trade | Natural gas amount (and impact) in 2004 is negative both for Import (due to Manufacture of rubber and plastic products (25)) and Export. In particular, Export-related impact in 2004 is significantly lower with regard to 2011 (+90%).  Natural gas impact increases for both Import and Export but Import-related impact is considerably higher (1.5 times) than Export-related one, thus leading to an overall increase of Consumption Footprint. |
|  | PL | POF:  +80% | +80% | Import | Increase of impact related to the Import (+31%). Main driver is the emission of NMVOCs to air, that increases by 36%, mostly linked to Manufacture of motor vehicles, trailers and semi-trailers (34) and Plastics from DE. |
|  |  | HTOX_c:  +52% | +52% | Domestic | Increase of formaldehyde emission to air (lack of data for years before 2005). |
|  |  | TEU:  -27% | -27% | Export | Increase of ammonia Export-related emission to air (+108% between 2004 and 2011). Linked activities: Cultivation of cereal grains nec and Processing of meat cattle. |
|  |  | AC:  -23% | -23% | Domestic | SOx emission due to Domestic activities reduced in 2011 with regard to 2004. The decrease is especially linked to public power, industry and shipping sectors. |
|  | LV | WU:  +16 | +16% | Import | Both Import- and Export-related impacts increase (+26% and +93% respectively) between 2004 and 2011.  Anyway, Import-related impact is much higher (almost 5 times) than Export-related one, thus leading the trend of Consumption Footprint. Main driver of Import water consumption in 2011 is the Fishing, operating of fish hatcheries and fish farms (to WM and LT). Main driver of Export is Processing of dairy products from LT. |
|  |  | MRU:  -80% | -80% | Import | Peak of gold traded (as both imported and exported) in 2004. The reduction until 2011 is quite significant (i.e. around -80%) for both the trade components. Still, the overall impact of import is considerably higher and its reduction is leading the global result.  Main drivers in gold input in 2011 are the production of chemicals and Manufacture of fabricated metal products, except machinery and equipment (28). |
|  |  | HTOX_nc:  -63% | -63% | Trade | Significant decrease of Import overall impact (-30%), led by a reduction in lead emission to air (-33%).  Main emission sector of lead to air in 2011 is Manufacture of basic iron and steel and of ferro-alloys and first products thereof from RU.  Slight increase of Export total impact (+8%), led by an increase in the emission of mercury to air (-20%).  Main emission sector of mercury to air in 2011 is Manufacture of fabricated metal products, except machinery and equipment (28). |
|  |  | LU:  -60% | -60% | Import | Impact from Import decreases (-69%). This is mainly due to a reduction in the use of Unspecified land (-76%).  Main sector leading the use of unspecified land in 2011 is Manufacture of wood and of products of wood and cork, except furniture; manufacture of articles of straw and plaiting materials (20). |
|  | LT | POF:  -67% | -67% | Trade | Decrease in the total impact linked to Import component (-22%) and considerable increase in the impact related to Export (+130%).  Both the trends are driven by NMVOC emissions. NMVOC impact due to Import reduces by -33%, whereas the NMVOC impact due to Export increases by +167%.  Leading sector in the emission of NMVOC for the Import component in 2011 is Manufacture of rubber and plastic products (25) from RU.  Leading sector in the emission of NMVOC for the Export component in 2011 is Plastics, basic to DE. |
|  |  | LU:  -64% | -64% | Trade | Impact from Import decreases (-53%). This is mainly due to a reduction in the use of Unspecified land (-65%).  Main sector leading the use of unspecified land in 2011 is Manufacture of wood and of products of wood and cork, except furniture; manufacture of articles of straw and plaiting materials (20).  Increase in Export-related impact (83%), mainly due to an increase in the use of unspecified land (+46%), forest land (+76%) and agriculture land (+215%).  Driving Export sector in the use of agriculture land in 2011 is Cultivation of wheat heading to WM. |
|  |  | AC:  -58% | -58% | Export | Increase of Export-related impact (+114% between 2004 and 2011) mainly driven by emission to air of ammonia (+157%), nitrogen oxides (+69%) and sulfur oxides (+60%).  The sectors driving the emission of these compounds in 2011 are respectively: Processing of meat cattle to RU and Petroleum Refinery to WM (for SOx). |
|  | EE | FRD:  +103% | +103% | Import | Imports’ impact increases by 212% mainly due to natural gas related to “hotels and restaurants” sector. |
|  |  | LU:  -83% | -83% | Imports | Decrease in imports mostly related to unspecified, forest (forestry & paper production) and agriculture (wheat, vegetable fats and oils, and meat pig) land uses. |
|  |  | MEU:  -68% | -68% | Imports | Decrease in imports of total nitrogen emissions to water (wheat, vegetable fats and oils, and meat pig) |
|  |  | TEU:  -60% | -60% | Import | Decrease in Import-related impact (-49%), due to a reduction in both ammonia (58%) and nitrogen oxides (-17%) emission to air.  Main emission sector for ammonia is Processing of meat cattle from LT and the one for NOx emission is Mining of nickel ores and concentrates from WA. |
| Stagnant | IT | FRD:  +24% | +24% | Import | Natural gas amount (and impact) in 2004 is negative both for Import and Export.  Anyway, Import-related total impact for this category is much higher (2.5 times) and its increase drives the overall Consumption Footprint.  Main sector responsible for the impact due to natural gas for Import in 2011 is Extraction of natural gas and services related to natural gas extraction, excluding surveying from WF. |
|  |  | FEU:  -24% | -24% | Import | Emission of phosphorus to water and soil reduce between 2004 and 2011, respectively by -14% and -13%.  Main emission sector for P to soil ant o water in 2011 is Processing of meat pigs from JP. |
|  | GR | AC:  -58% | -58% | Domestic | Decrease in domestic emissions of sulfur oxides (solvents, road transport, international shipping, agriculture, shipping, energy sector). |
|  |  | POF:  -48% | -48% | Trade and domestic | Increase in exports (+31%) of most of emissions due to sea and coastal water transport (CN, US, JP, KR). Also, decrease in imports (-20%) and in domestic NOx emissions (-30%) (industry, solvents, road transport, agriculture, international shipping). |
|  |  | MRD:  +55% | +55% | Import | Increase in imports’ impact (+56%) mainly due to Mining of precious metal ores and concentrates (RU). |
|  |  | LU:  +15% | +15% | Import | Increase of grassland occupation in imports [Processing of meat cattle (AU, WL), and Processing vegetable oils and fats (WM)]. |

# ESM 4. Contribution of midpoint categories to weighted score (2004-2011).

The following tables shows the contribution of the midpoint categories to the weighted score along the period 2004-2011 for the bottom-up approach (Table 4.A) and the top-down approach (Table 4.B).

**Table 4.A.** Consumption footprint top-down: Contribution of the impact categories to the weighted score.

| **Category** | **2004** | **2005** | **2006** | **2007** | **2008** | **2009** | **2010** | **2011** |
| --- | --- | --- | --- | --- | --- | --- | --- | --- |
| HTOX_c | 2% | 2% | 3% | 2% | 2% | 2% | 2% | 2% |
| HTOX_nc | 2% | 3% | 3% | 2% | 2% | 2% | 1% | 2% |
| PM | 16% | 17% | 16% | 17% | 16% | 15% | 16% | 14% |
| POF | 4% | 4% | 4% | 4% | 4% | 4% | 4% | 3% |
| WU | 6% | 6% | 6% | 6% | 6% | 6% | 6% | 6% |
| ECOTOX | 1% | 1% | 1% | 1% | 1% | 1% | 1% | 1% |
| CC | 22% | 23% | 23% | 23% | 22% | 21% | 22% | 20% |
| FRD | 7% | 12% | 16% | 12% | 13% | 13% | 13% | 11% |
| MEU | 2% | 2% | 2% | 2% | 2% | 2% | 2% | 2% |
| FEU | 2% | 2% | 2% | 2% | 1% | 1% | 1% | 1% |
| LU | 5% | 5% | 5% | 5% | 5% | 5% | 5% | 5% |
| TEU | 4% | 4% | 4% | 4% | 4% | 4% | 4% | 3% |
| AC | 7% | 7% | 7% | 7% | 7% | 6% | 6% | 6% |
| MRD | 20% | 12% | 10% | 12% | 14% | 19% | 18% | 23% |

**Table 4.B.** Consumption footprint bottom-up: Contribution of the impact categories to the weighted score.

| **Category** | **2005** | **2010** |
| --- | --- | --- |
| HTOX_c | -1% | -3% |
| HTOX_nc | 1% | 0% |
| PM | 19% | 20% |
| POF | 5% | 5% |
| WU | 6% | 6% |
| ECOTOX | 2% | 2% |
| CC | 26% | 28% |
| FRD | 18% | 19% |
| MEU | 3% | 3% |
| FEU | 2% | 2% |
| LU | 3% | 3% |
| TEU | 4% | 5% |
| AC | 9% | 9% |
| MRD | 2% | 1% |

# ESM 5. Trends of midpoint categories (2005-2014).

The following figures shows the decoupling at the midpoint category level for the bottom-up approach (Figure 5.A) and the top-down approach (Figure 5.B).

**Figure 5.A.** Decoupling of the 15 midpoint categories (HTOX_c is excluded due to negative values) from the economic activity (GDP) for the CF-BU (2005-2014).

**Figure 5.A.** Decoupling of the 14 midpoint categories from the economic activity (GDP) for the CF-TD (2005-2014).

# ESM 6. Life cycle parameters: Decoupling assessment at the country level (EU-28 normalization).

This section details the decoupling assessment at the country level by employing the EU-28 normalization instead of the global one. To contextualize the change of the normalization, the share of the global impacts for 2010 (Crenna et al., 2019) that are attributed to the EU-28 area are shown (Table 5.A). Second, Figure 6.A shows the decoupling of the Consumption footprint top-down (CF-TD) at the country level (2004-2011, EU-28 normalization). Finally, Figure 6.B displays the comparison of assessing the decoupling from the economic activity (GDP) and the human well-being (HDI).

**Table 6.A.** Share of the EU-28 total impact regarding the global impact (2010), by impact category.

| **Impact category** | **Unit** | **2005** | **2006** | **2007** | **2008** | **2009** | **2010** | **2011** | **2012** | **2013** | **2014** |
| --- | --- | --- | --- | --- | --- | --- | --- | --- | --- | --- | --- |
| HTOX_c | CTUh | 5% | 5% | 5% | 5% | 5% | 5% | 5% | 5% | 5% | 5% |
| HTOX_nc | CTUh | 4% | 4% | 4% | 3% | 3% | 3% | 3% | 3% | 3% | 3% |
| PM | Disease incidence | 13% | 13% | 13% | 13% | 12% | 12% | 12% | 12% | 11% | 11% |
| POF | kg NMVOC eq | 7% | 7% | 7% | 6% | 6% | 6% | 5% | 5% | 5% | 5% |
| IR | kBq U^235^ eq | 76% | 69% | 60% | 54% | 62% | 63% | 59% | 63% | 67% | 64% |
| WU | m^3^ world eq | 5% | 5% | 5% | 5% | 5% | 5% | 5% | 5% | 5% | 5% |
| ECOTOX | CTUe | 5% | 5% | 5% | 5% | 5% | 5% | 5% | 5% | 5% | 5% |
| CC | kg CO_2_ eq | 10% | 10% | 9% | 9% | 8% | 9% | 8% | 8% | 8% | 8% |
| FRD | MJ | 7% | 7% | 7% | 6% | 6% | 6% | 6% | 6% | 5% | 5% |
| ODP | kg CFC-11 eq | 3% | 3% | 3% | 3% | 3% | 3% | 2% | 2% | 2% | 2% |
| MEU | kg N eq | 7% | 7% | 7% | 7% | 6% | 6% | 6% | 7% | 6% | 6% |
| FEU | kg P eq | 6% | 5% | 5% | 5% | 5% | 5% | 4% | 5% | 4% | 4% |
| LU | Pt | 3% | 3% | 3% | 3% | 3% | 3% | 3% | 3% | 3% | 3% |
| TEU | molc N eq | 9% | 9% | 8% | 8% | 8% | 8% | 8% | 7% | 7% | 7% |
| AC | molc H^+^ eq | 8% | 8% | 8% | 7% | 7% | 6% | 6% | 6% | 6% | 6% |
| MRD | kg Sb eq | 2% | 2% | 2% | 2% | 2% | 2% | 2% | 2% | 2% | 2% |
| Single (14) | - | 7.3% | 7.3% | 7.0% | 6.8% | 6.4% | 6.6% | 6.2% | 6.3% | 6.0% | 6.0% |
| Single (16) | - | 10.5% | 10.1% | 9.4% | 8.9% | 9.0% | 9.2% | 8.6% | 8.8% | 8.8% | 8.7% |

**Figure 6.A**. Decoupling at the country level (2004-2011): Decoupling of the CF-TD from the economic activity (GDP) and the human well-being (HDI). Bullet size represents country population (2010) (Eurostat, 2018b).
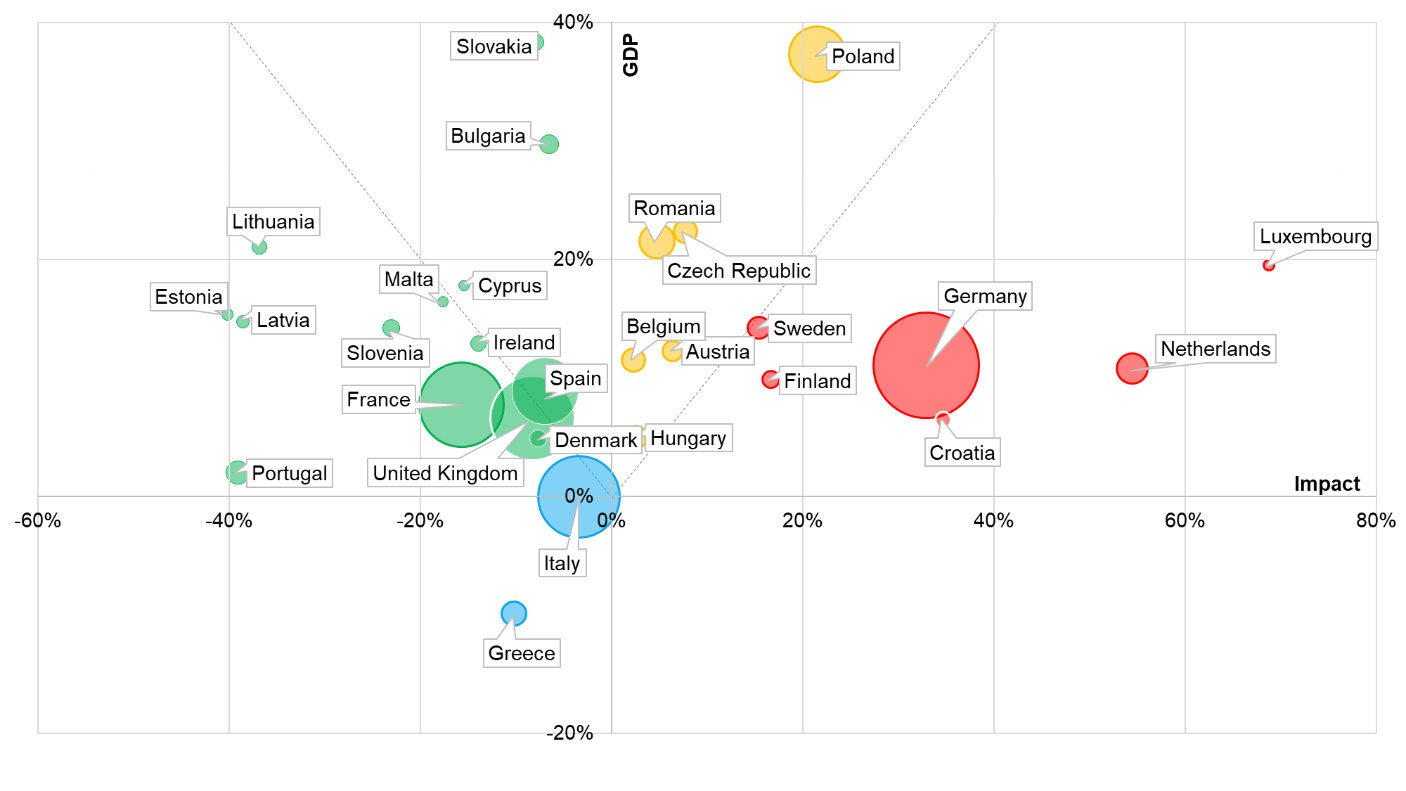


**
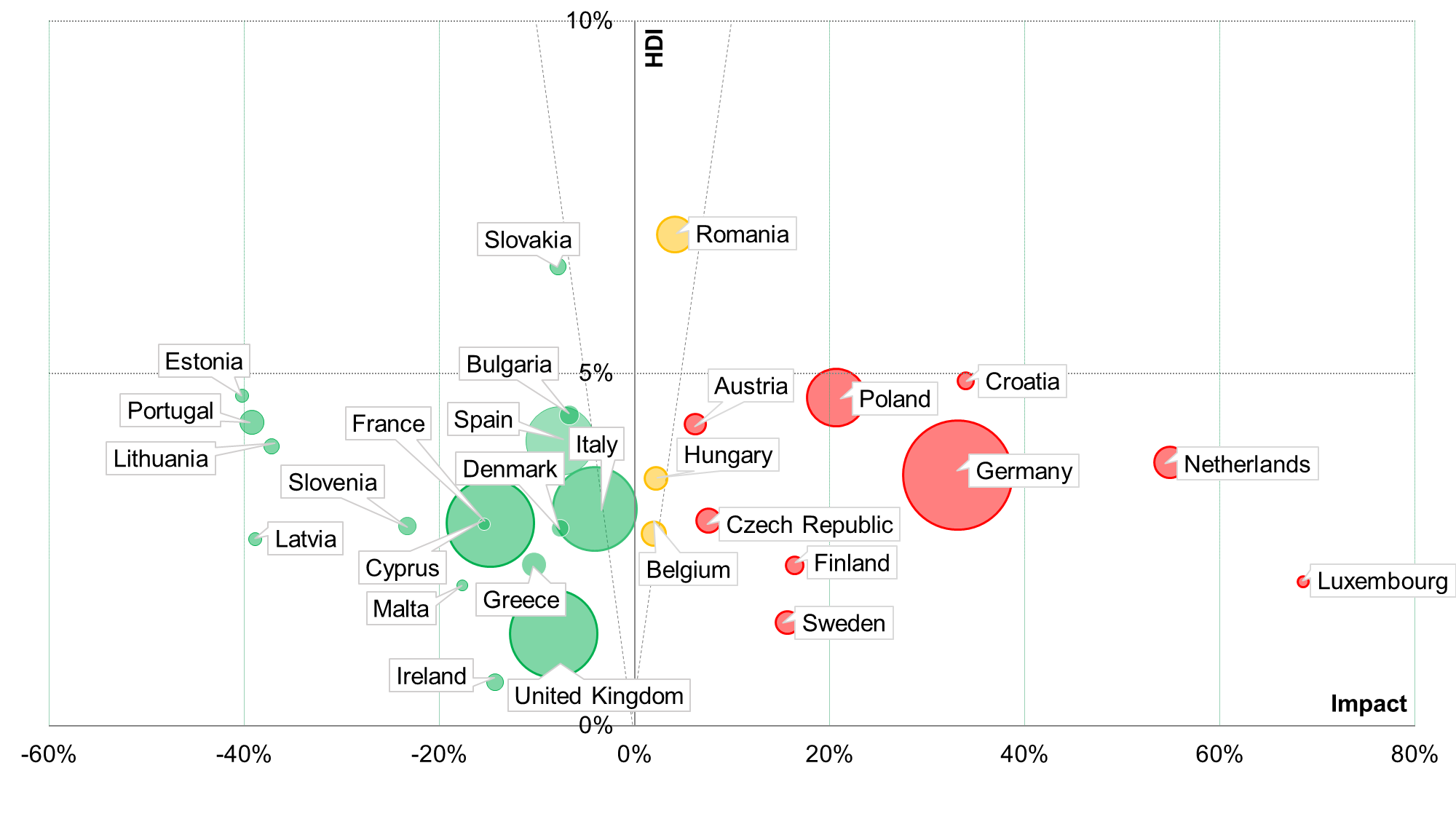
**

# ESM 7. Drivers of Consumption Footprint top-down decoupling at the country level: group examples (EU-28-normalised).

Examples of each decoupling group (i.e., absolute decouplers, relative decouplers, non-decouplers and stagnant) were evaluated to determine the drivers of the largest changes in the environmental impacts of individual countries. The identification of the consumption element, the flows, the economic sectors and the products were evaluated based on the top-down analysis of trade and the flows inventory of the domestic element.

**Absolute decouplers: Slovakia, Lithuania and Estonia.** A decreased production of electricity from non-renewable sources (nuclear power, fossil fuels), a decreased trade of manufactured products with electronic parts (e.g., vehicles) and of bio-based products (e.g., meat), as well as a variation in the PM_2.5_ emissions motivated the decreasing trends in the CF-TD.

**Relative decouplers: Poland.** A decreased export of food products and data gaps in the initial years (e.g. photochemical ozone formation and CO and CH_4_ emissions) originated the main variations of the CF-TD.

**Stagnant countries: Greece and Italy.** Reductions are mainly related to a variation in the role of fossil fuels in the energy mix of Italy, where renewable energy is growing, and variation in water impacts related to food imports. The decrease of the Consumption Footprint of Greece is associated to reduced domestic emissions of SOx and NOx (e.g. solvents, road transport, international shipping) and the environmental impacts embodied in the exports related to the sea and coastal water transport sector.

**Non-decouplers: Luxemburg (LU), The Netherlands (NL) and Germany (DE).** An increased meat trade (LU, NL), enlarged embodied burdens of the ‘hotel and restaurant’ and manufactured products trade (LU), and the increased imports of gold and other industrial minerals (DE) and a low decrease of grassland land use compared to the rest of EU-28 (DE) were the main drivers towards a non-decoupling consumption.

**Table 7.A.** Explanation of outliers in the Consumption Footprint top-down decoupling (2004-2011) for 14 indicators, by country: category variation (weighted and characterized value), consumption element and explanation.

|  | **Country** | **Category** | | | **Consumption**  **element** | | **Explanation** |
| --- | --- | --- | --- | --- | --- | --- | --- |
|  |  | **(We. var.)** | **(Ch. var.)** |  | |  | |
| Non- decouplers | LU | HTOX_c:  +416% | 431% | Imports | | Increase of chromium emissions to air in imports due to trade of metal manufactured products (steel, iron, metal) from CN, NL and RU. | |
|  |  | MRD:  +163% | 192% | Imports | | Increased imports of gold for chemicals nec (WA, WL) and radio equipment (WM) and imported tin from BR and WL. | |
|  |  | FRD:  -226% | -381% | Imports | | In 2004, both trade elements have a negative impact. Data from Exiobase indicate there are negative flows of natural gas due to the economic sectors Production of electricity by biomass and waste, Manufacture of motor vehicles, trailers and semi-trailers (34), and Manufacture of rubber and plastic products (25). | |
|  |  | *HTOX_nc:*  *-212%* | -194% | Imports  & Exports | | Increase of mercury and arsenic emissions to air in both trade elements, embodied in imported manufactured metal products from RU, NL and CN; and exported to FR, DE and NL. | |
|  | NL | TEU:  +167% | +86% | Imports | | Increase in imported ammonia emissions to air due to imports of meat (cattle, pigs and poultry) (WL, GB, DE, WM). | |
|  |  | FEU:  +147% | +67% | Exports | | Decrease of exports’ impact and the subsequent P emissions to soil related to meat production (processing and pig farming) to DE. | |
|  |  | MEU:  +125% | +76% | Imports | | Increase in imports and the embodied N emissions to water due to meat (pigs) (DE), meat cattle (GB), cereals (FR) and meat poultry (WM). | |
|  |  | AC:  +127% | +50% | Trade | | Increase in imports and decrease in exports, leading to a higher contribution of ammonia emissions to air, mainly due to imports of meat (cattle, pigs and poultry) (WL, GB, DE, WM). | |
|  | DE | LU:  +9% | -10% | Imports | | Decrease in land use of Germany was lower than the EU-28 average. Main decrease is shown by grassland land use, linked to imports of hotels and restaurants services and imported meat. | |
|  |  | MRD:  +5% | +38% | Imports | | Increase in imports’ impact due to increase of the depletion of gold [Chemicals nec (WL), Mining of precious metal ores and concentrates (MX) and Precious metals production (RU)] and other industrial minerals [Construction (45) and Chemicals nec from China]. | |
|  |  | FRD:  -116% | -135% | Trade | | Decrease in exports due to uranium, and increase in imports. Regarding imports, even crude oil has decreased in this period, there is an increase in the impact related to natural gas as this was negative in 2004 (manufactured products: Manufacture of motor vehicles, trailers and semi-trailers (34), Manufacture of rubber and plastic products (25), Manufacture of office machinery and computers (30), Manufacture of furniture; manufacturing n.e.c. (36). Current natural gas is imported from Middle East and Norway. | |
| Relative | PL | FRD:  +384% | +30% | Domestic  and trade | | Decrease in hard coal consumption (domestic), resulting in a higher import of crude oil. As well, the export of other bituminous coal decreases (related to Mining of coal and lignite; extraction of peat (WE), Production of electricity by coal (DE) and Manufacture of basic iron and steel and of ferro-alloys and first products thereof (WM)). | |
|  |  | POF:  +153% | +80% | Domestic | | Increase in domestic emissions of CO and CH4, although there are some data gaps for some substances. | |
|  |  | MEU:  -23% | -11% | Exports | | Increase of total N emissions to water embodied in exports of vegetables and cereal products to DE. | |
|  |  | TEU:  -25% | -27% | Exports | | Increase of NH_3_ emissions to air embodied in exports of cereals (DE) and meat (TR, IT). | |
| Absolute | SK | HTOX_c:  -54% | -66% | Trade | | Decrease of imports and exports, declining chromium emissions from manufactured products (e.g. medical, precision and optical instruments, watches and clocks, radio, television and communication equipment and apparatus, furniture, motor vehicles, machinery and equipment). | |
|  |  | HTOX_nc:  -43% | -57% | Trade | | Decrease of imports and exports, reducing the emissions of lead and mercury from manufactured products (e.g. medical, precision and optical instruments, watches and clocks, radio, television and communication equipment and apparatus, furniture, motor vehicles, machinery and equipment). | |
|  |  | AC:  +59% | +6% | Imports | | Increase of imported NH_3_ emissions (Dairy and meat products form CZ, IT, NL, HU) and SOx emissions (import of manufactured products – vehicles from DE, CN, FR, RU and machinery from DE). | |
|  |  | TEU:  +56% | +5% | Imports | | Increase of imported NH_3_ emissions (Dairy and meat products form CZ, IT, NL, HU) and NOx emissions (import of manufactured products – vehicles from DE, CN, FR, RU and machinery from DE). | |
|  |  | MEU:  +55% | +9% | Imports | | Increase of imported N emission to water originated in imports of meat pigs (US, CZ, CA), meat cattle (CZ) and vegetables oils and fats (CZ). | |
|  | LT | FRD:  -70% | -37% | Trade  and domestic | | Decrease of imports of crude oil (WM, RU, WL) and increase in its exports (petroleum refinery: WM, US, PL, FR, CA). As well, there is a decrease in domestic uranium depletion. | |
|  | EE | AC:  -92% | -58% | Trade | | Decrease of NH_3_ emission embodied in imports, mostly related to meat cattle, N-fertilisers and hotels and restaurants.  Increase of SOx emissions embodied in exported electricity (FI, LV, RU) and pulp (IN). | |
|  |  | PM:  -86% | -3% | Domestic | | Domestic increase of PM_2.5_ emissions (increase of the different sectors – public power, road transport, aviation, agriculture, international shipping). | |
|  |  | LU:  -85% | -83% | Imports | | Decrease in imports mostly related to unspecified, forest (forestry & paper production) and agriculture (wheat, vegetable fats and oils, and meat pig) land uses. | |
|  |  | MEU:  -65% | -68% | Imports | | Decrease in imports of total nitrogen emissions to water (wheat, vegetable fats and oils, and meat pig) | |
| Stagnant | IT | FRD:  -37% | +24% | Export | | Notwithstanding the increase of the characterised impact, this is lower than the EU-28 average. Increase in export burdens (+38%), due to a negative impact linked to natural gas consumption in 2004. | |
|  |  | WU:  +12% | +1% | Imports | | Decrease in import consumption of freshwater, mainly related to imported vegetable oils and fats (WF) and meat pig (JP). | |
|  | GR | FEU:  -1150% | -23% | Domestic | | Decrease in domestic emissions of Phosphorous to water, related to wastewater treatment. | |
|  |  | AC:  -108% | -58% | Domestic | | Decrease in domestic emissions of sulphur oxides (solvents, road transport, international shipping, agriculture, shipping, energy sector). | |
|  |  | LU:  +83% | +15% | Imports | | Increase of grassland occupation in imports [Processing of meat cattle (AU, WL), and Processing vegetable oils and fats (WM)]. | |

# ESM 8. Temporal scope: Annual decoupling

The following table represents the annual EU-28 decoupling for the time frame under assessment (2005-2014) by impact category and for the weighted score of the Consumption footprint top-down. Decoupling is classified as absolute decoupling (green), relative decoupling (yellow) and non-decoupling (red).

**Table 8.A.** Annual decoupling of EU-28 consumption for the timeframe 2005-2014 for the 14 impact categories under assessment and the weighted score (global normalization).

| **PERCENTAGE** | **2005** | **2006** | **2007** | **2008** | **2009** | **2010** | **2011** | **2012** | **2013** | **2014** |
| --- | --- | --- | --- | --- | --- | --- | --- | --- | --- | --- |
| HTOX_c | 4,7 | 2,5 | -1,2 | -7,9 | 6,2 | 1,5 | 9,6 | -12,7 | 14,3 | 2,5 |
| HTOX_nc | 6,0 | 2,1 | -8,4 | -30,3 | 0,8 | -24,6 | 82,1 | -9,6 | 13,7 | 2,6 |
| PM | 1,8 | -1,8 | 1,4 | -3,0 | 3,1 | 3,5 | -4,3 | -1,0 | -3,0 | -1,9 |
| POF | 1,3 | -3,0 | -0,4 | 4,8 | 3,3 | 2,4 | -2,5 | 2,7 | -8,2 | -1,4 |
| WU | 0,3 | -2,6 | 0,3 | 11,6 | 1,3 | -0,3 | 2,1 | 0,0 | -5,8 | -1,0 |
| ECOTOX | 0,6 | 0,5 | -0,2 | -3,2 | 0,9 | -1,0 | 1,9 | -0,6 | -1,6 | -0,2 |
| CC | 1,8 | -0,3 | -0,6 | -1,3 | 2,6 | 2,6 | -2,3 | 1,9 | -5,4 | -1,5 |
| FRD | 35,9 | 11,8 | -9,4 | 34,1 | 2,5 | 2,9 | -6,7 | 6,3 | -7,9 | -1,6 |
| MEU | 0,4 | -3,8 | 2,2 | -3,6 | 2,0 | 0,1 | -1,0 | -2,8 | -16,5 | -0,9 |
| FEU | -0,5 | -3,0 | -0,8 | 2,5 | 2,6 | 3,6 | -5,3 | -13,3 | -11,6 | -1,6 |
| LU | -1,6 | -2,2 | -0,8 | -6,7 | 1,8 | 1,6 | 3,5 | 1,8 | -2,7 | -0,5 |
| TEU | 0,1 | -3,5 | 3,9 | -14,4 | 2,8 | 5,0 | -7,7 | 4,2 | -5,5 | -0,7 |
| AC | 0,4 | -3,4 | 2,6 | -13,2 | 3,0 | 1,4 | -4,6 | 5,3 | -8,5 | -0,7 |
| MRD | -19,3 | -5,5 | 4,5 | 63,3 | -4,9 | 0,6 | 15,5 | -16,1 | 25,5 | 3,9 |
| **Weighted score** | -0,5 | -0,5 | -1,0 | 8,7 | 1,6 | 1,6 | 1,3 | -2,8 | 2,5 | 0,2 |

# ESM 9. Temporal scope: Domestic footprint decoupling (2000-2014; 2005-2014, 2004-2011)

The following table and figures compare the decoupling assessment for the domestic footprint for different temporal scopes. Table 9.A compares the results between 2000 and 2014, and the assessment period (2005-2014). Figure 9.A shows the decoupling behavior by country in relation to the variation of the economic output and the environmental impact for the period 2000-2014 and the period 2004-2011, where data at country level is the most robust. In general, longer periods show a higher amount of absolute decouplers as economic output is constantly growing in a capitalistic-minded economic structure and, thus, it is more probable that the economic output growth offsets the environmental impact increase.

**Table 9.A.** Comparison of decoupling results for the domestic footprint (14 indicators, global normalized) for two different time periods: 2005-2014 and 2000-2014.

| **Ind.** | **2005-2014** | **2000-2014** |
| --- | --- | --- |
| **14** | 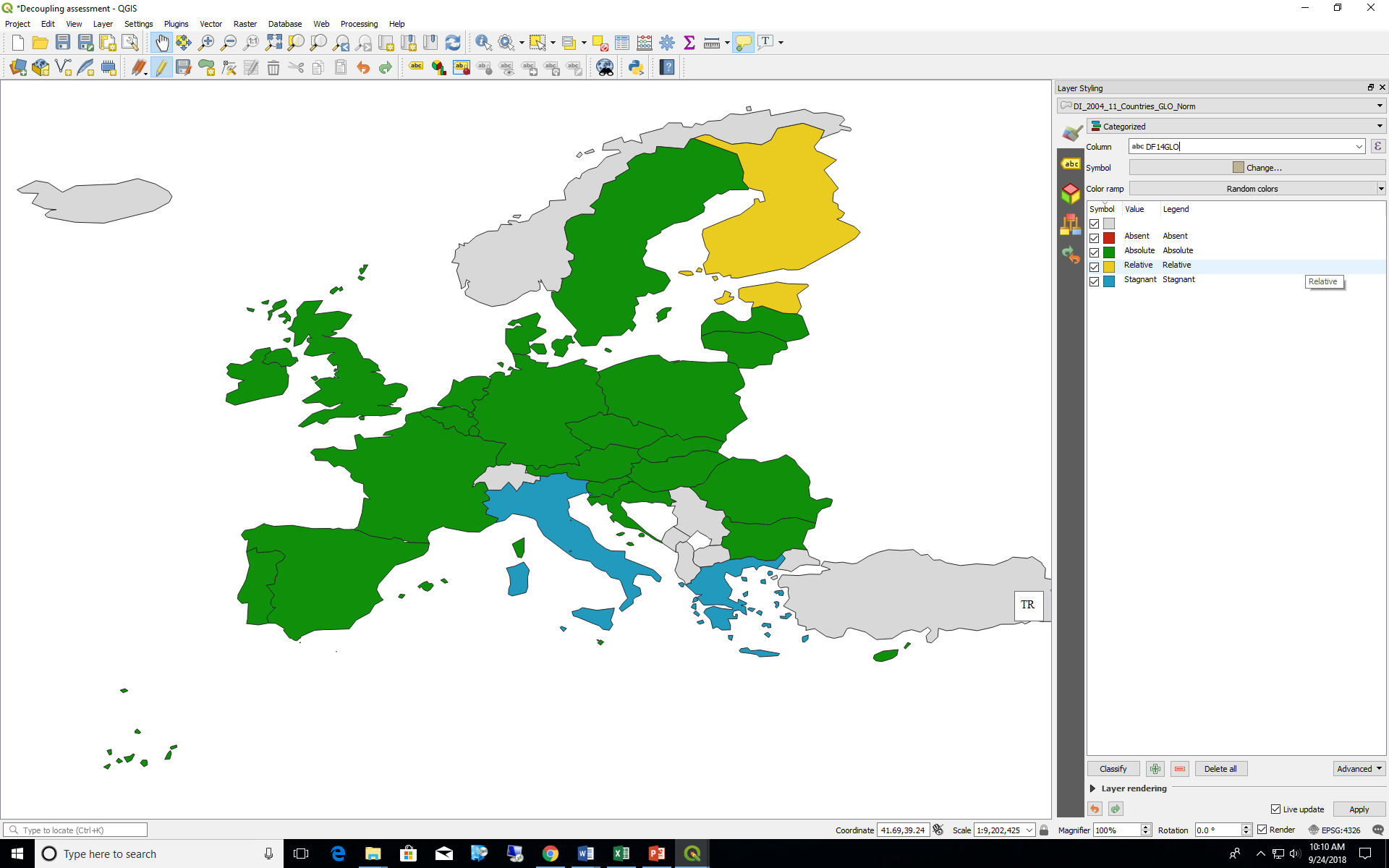 | 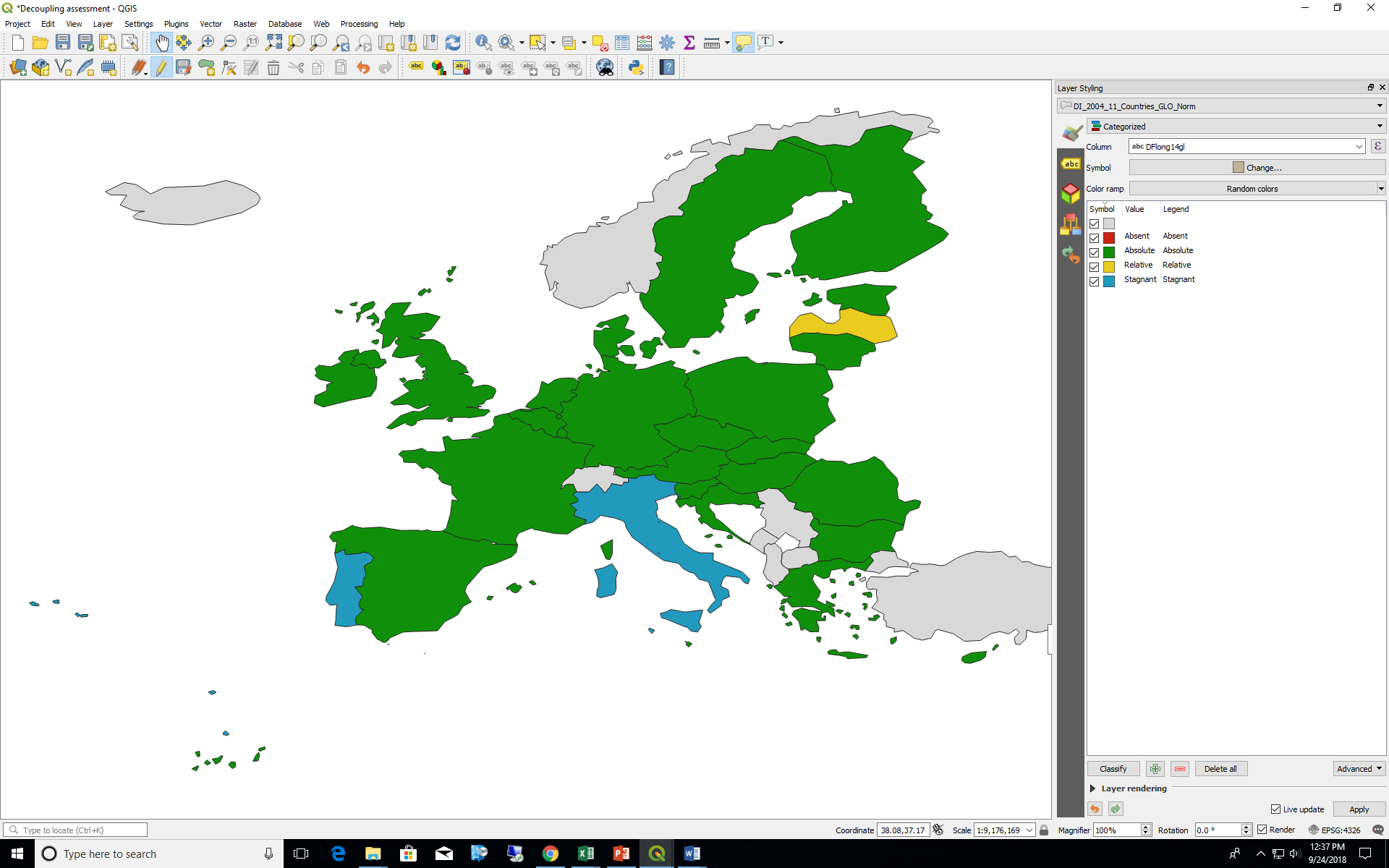 |

**Figure 9.A.** Comparison of decoupling results for the domestic footprint (14 indicators, global normalized) for two different time periods: 2005-2014 and 2000-2014.


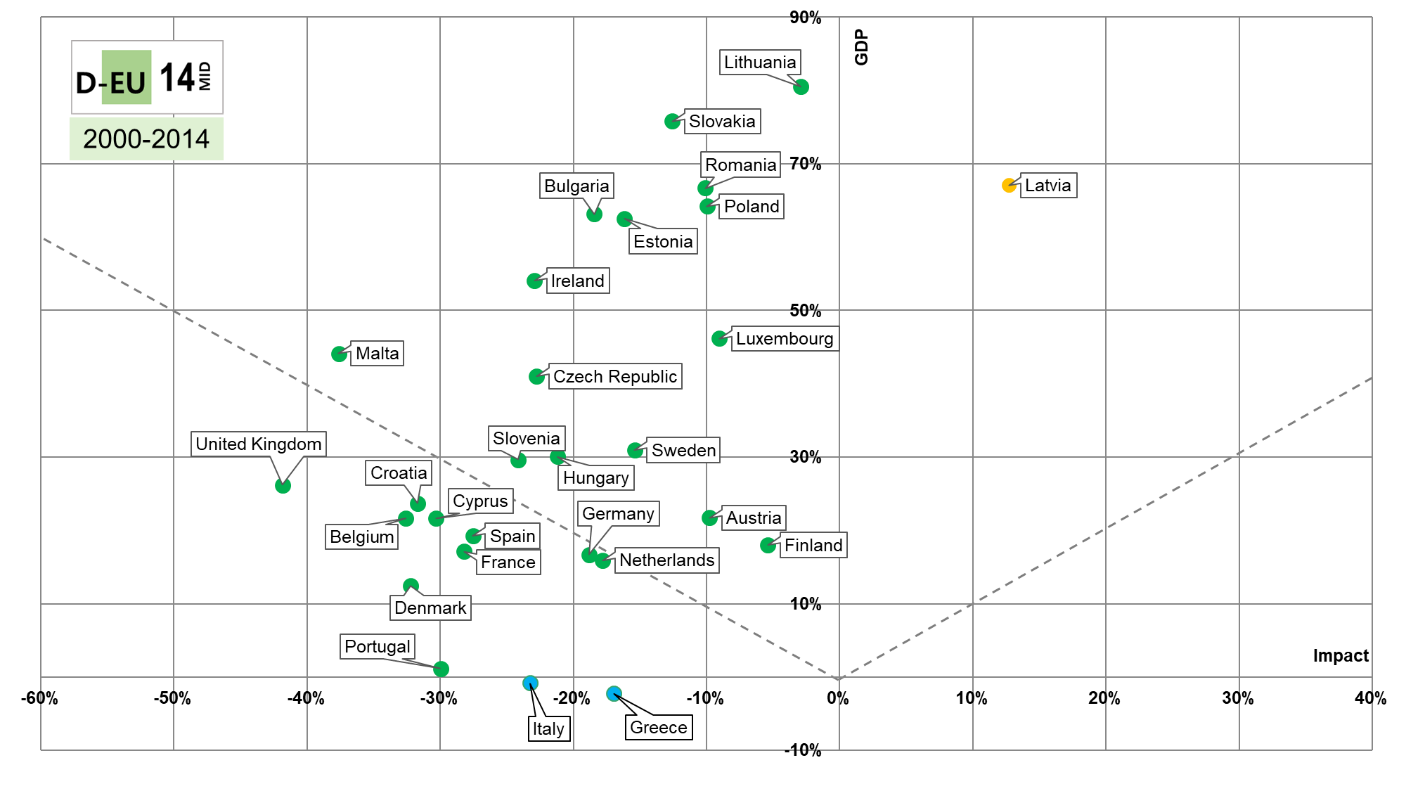


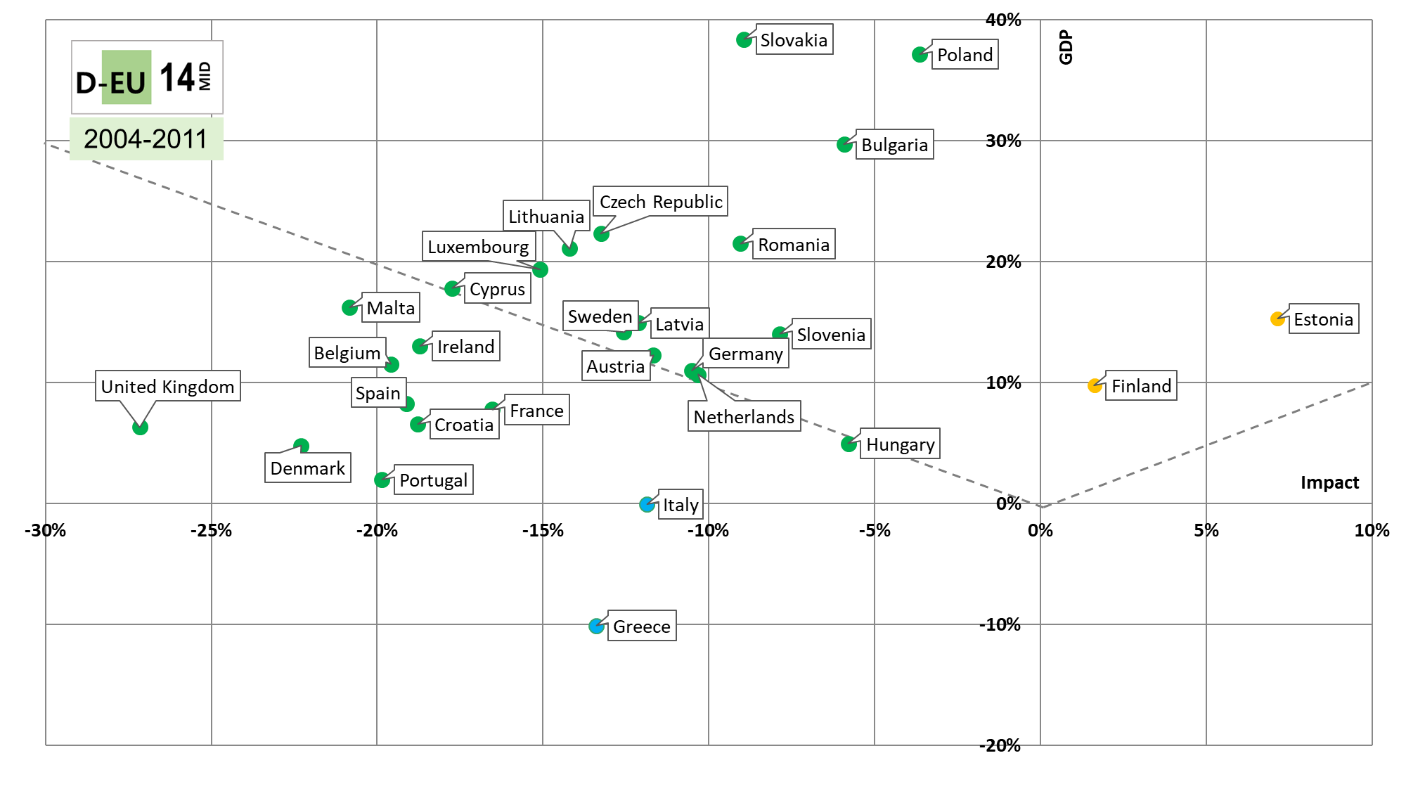


# References

Crenna, E., Secchi, M., Benini, L., & Sala, S. (2019). Global environmental impacts: data sources and methodological choices for calculating normalization factors for LCA. The International Journal of Life Cycle Assessment, 1-27.

EC. (2017). PEFCR Guidance document - Guidance for the development of Product Environmental Footprint Category Rules (PEFCRs), version 6.3, December 2017. Retrieved from http://ec.europa.eu/environment/eussd/smgp/pdf/PEFCR_guidance_v6.3.pdf

Eurostat. (2018a). Domestic material consumption - tonnes per capita. Retrieved from http://ec.europa.eu/eurostat/data/database

Eurostat. (2018b). Population on 1 January by age and sex (demo_pjan). Retrieved from http://ec.europa.eu/eurostat/data/database
